# Coupling of nanocrystal hexagonal array and two-dimensional metastable substrate boosts H_2_-production

**DOI:** 10.1038/s41467-022-33512-5

**Published:** 2022-10-03

**Authors:** Zhenglong Fan, Fan Liao, Yujin Ji, Yang Liu, Hui Huang, Dan Wang, Kui Yin, Haiwei Yang, Mengjie Ma, Wenxiang Zhu, Meng Wang, Zhenhui Kang, Youyong Li, Mingwang Shao, Zhiwei Hu, Qi Shao

**Affiliations:** 1 Institute of Functional Nano & Soft Materials (FUNSOM), Jiangsu Key Laboratory for Carbon-Based Functional Materials & Devices, Soochow University, Suzhou, 215123 Jiangsu China; 2 College of Chemistry, Chemical Engineering and Materials Science, Soochow University, Suzhou, 215123 Jiangsu China; 3 College of Energy, Soochow University, Suzhou, 215123 Jiangsu China; 4 Macao Institute of Materials Science and Engineering, Macau University of Science and Technology, Taipa, 999078 Macau SAR China; 5 Max Planck Institute for Chemical Physics of Solids, Nothnitzer Strasse 40, Dresden, 01187 Germany

**Keywords:** Metamaterials, Two-dimensional materials, Electrocatalysis

## Abstract

Designing well-ordered nanocrystal arrays with subnanometre distances can provide promising materials for future nanoscale applications. However, the fabrication of aligned arrays with controllable accuracy in the subnanometre range with conventional lithography, template or self-assembly strategies faces many challenges. Here, we report a two-dimensional layered metastable oxide, trigonal phase rhodium oxide (space group, P-3m1 (164)), which provides a platform from which to construct well-ordered face-centred cubic rhodium nanocrystal arrays in a hexagonal pattern with an intersurface distance of only 0.5 nm. The coupling of the well-ordered rhodium array and metastable substrate in this catalyst triggers and improves hydrogen spillover, enhancing the acidic hydrogen evolution for H_2_ production, which is essential for various clean energy-related devices. The catalyst achieves a low overpotential of only 9.8 mV at a current density of −10 mA cm^−**2**^, a low Tafel slope of 24.0 mV dec^−**1**^, and high stability under a high potential (vs. RHE) of −0.4 V (current density of ~750 mA cm^−**2**^). This work highlights the important role of metastable materials in the design of advanced materials to achieve high-performance catalysis.

## Abstract (web summary)

The construction of well-ordered nanoarrays, particularly invoking metastable material phases, remains a challenge. Here, authors prepared a well-ordered, rhodium nanoarray on two-dimensional, metastable rhodium oxide to enhance hydrogen evolution electrocatalysis.

## Introduction

The development of highly efficient electrochemical catalysts for the hydrogen evolution reaction (HER) through water splitting is a critical step in the advancement of hydrogen production for energy storage and conversion in modern industry^1–6^. Furthermore, the simple HER is a less sophisticated process in terms of understanding the mechanism of the water catalytic reaction and the relationship between the electrocatalytic activity and crystal structure at the nanoscale than the four-step oxygen evolution reaction/oxygen reduction reaction processes. According to Trassati’s volcano plot, rhodium (Rh) or Rh-based materials are promising catalysts for the HER^7–9^. However, the adsorption energy of hydrogen (ΔG_H_) on the Rh surface is still relatively high, which is unfavourable for the formation of H_2_. In addition, the poor durability of these materials makes it necessary to design structures to achieve enhanced HER performance^9^.

It is well known that nanosized entities in periodic identical arrays strongly influence the electronic and transport properties of the material, providing collective characteristics different from those of the corresponding bulk structures^10,11^. To date, many periodic nanostructures have been reported, showing promise in applications in energy conversion, catalysis, and photoelectronic devices^12–17^. Notably, a perfectly aligned nanocrystal array with an interparticle distance of a few nanometres may provide a platform for pursuing different catalytic properties. However, the traditional nanolithography and template methods employed to fabricate the aligned array assembly always yield interparticle distances larger than 10 nm^18^. Thus, developing a strategy to fabricate a perfectly aligned nanocrystal array with a short interparticle distance (less than 5 nm) is highly desirable.

Two-dimensional (2D) metastable metal oxides may provide an ideal substrate for overcoming the above challenges. 2D materials have attracted extensive attention due to their maximum atomic utilization, ideal activities and desirable durability^19–32^. In addition, metastable metal oxides provide extensive possibilities for synthesizing the interfacial structures due to their intrinsic metastable properties^33–35^. Furthermore, in the metal/oxide catalytic interfacial system, the hydrogen spillover effect can cause the activated hydrogen atoms to migrate from a hydrogen-rich area to a hydrogen-poor area, which may provide an effective way to further improve the HER activity^36,37^.

In this work, we report a perfectly aligned nanocrystal array on a pristine 2D metastable trigonal rhodium oxide (P-Tri-RhO_2_). P-Tri-RhO_2_ was fabricated by a radiofrequency-assisted molten-alkali method, and its crystal structure is categorized as space group P-3m1 (164), with lattice constants of *a* = *b* = 3.091 Å and *c* = 4.407 Å. More importantly, the 0.45% lattice mismatch between metastable P-Tri-RhO_2_ and face-centred cubic (fcc) phase Rh leads to the in situ growth of Rh single-crystal nanoarrays with a short interparticle spacing of 3.709 nm. Such nanoscale spacing enables the spillover of hydrogen atoms to greatly enhance the HER with an ultralow overpotential of 9.8 mV at a current density of −10 mA cm^−2^, a low Tafel slope of 24.0 mV dec^−1^ and limited activity decay under a high potential (vs. RHE) of −0.4 V.

## Results

### Preparation and structure characterization of P-Tri-RhO_2_

**Fig. 1:**
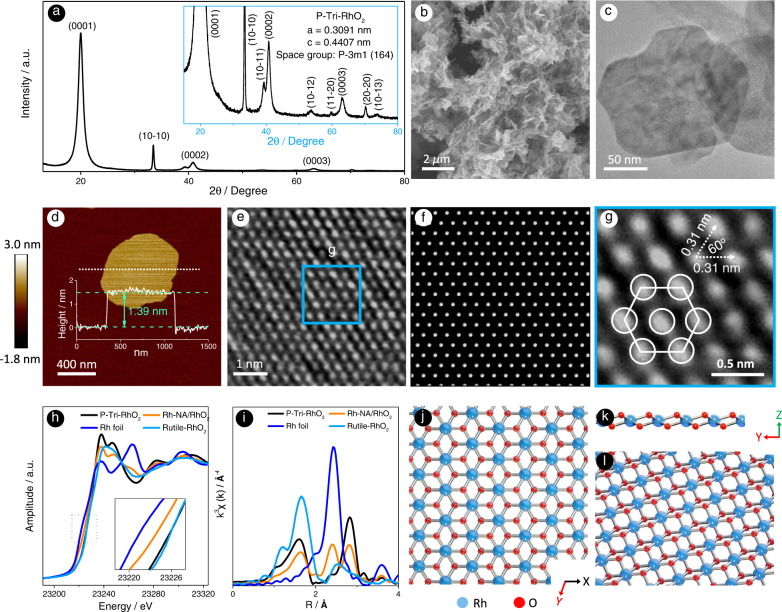
Structure characterization of P-Tri-RhO_2_ and Rh-NA/RhO_2_. **a** XRD pattern and its enlarged view. **b** The SEM and (**c**) TEM images, showing its 2D nanosheets morphology. **d** AFM image and the corresponding height profile. **e** STEM image, clearly showing the atomic arrangement of Rh. **f** Simulated STEM image and (**g**) partial enlargement from (**e**). **h** Normalized Rh K-edge XANES spectra for P-Tri-RhO_2_, Rh-NA/RhO_2_, Rh foil and standard Rutile-RhO_2_. **i** Normalized Fourier transformed (FT) k^3^-weighted χ (k)-function of the extended X-ray absorption fine structure (EXAFS) spectra for P-Tri-RhO_2_, Rh-NA/RhO_2_, Rh foil and standard Rutile-RhO_2_ reference at Rh K-edge. **j**–**l** The corresponding atomic models of P-Tri-RhO_2_ from different directions.

Pristine trigonal RhO_2_ (P-Tri-RhO_2_) was synthesized via a radiofrequency assisted molten-alkali method (Supplementary Fig. 1), where rhodium (III) chloride (RhCl_3_) and potassium hydroxide (KOH) were selected as the raw materials. The final product of P-Tri-RhO_2_ is brown (Supplementary Fig. 2a). Scanning electron microscopy (SEM) and transmission electron microscopy (TEM) were applied to characterize the morphology of P-Tri-RhO_2_, revealing its 2D ultrathin nanosheet morphology (Fig. 1b, c and Supplementary Fig. 2b). The selected area electron diffraction (SAED) patterns of the P-Tri-RhO_2_ sheet are hexagonal (Supplementary Fig. 2c), consistent with its trigonal structure. Atomic force microscopy (AFM) images reveal that the thickness of P-Tri-RhO_2_ is approximately 1.39 nm (Fig. 1d). The crystal structure of P-Tri-RhO_2_ is first revealed by X-ray diffraction (XRD), as shown in Fig. 1a. The crystal parameters are determined to be *a* = *b* = 3.091 Å and *c* = 4.407 Å. In addition, the simulated XRD pattern of P-Tri-RhO_2_ is almost the same as the XRD pattern of P-Tri-RhO_2_, further confirming its trigonal phase (Supplementary Fig. 3). Energy dispersive X-ray spectroscopy (EDX) results reflect that Rh and O are uniformly distributed in P-Tri-RhO_2_ and that there is no K signal (Supplementary Fig. 4a–e). The elemental analysis results (elementar EL III) suggest that the atomic ratio of Rh and O is approximately 1:2 (Supplementary Fig. 4f). The Brunauer‒Emmett‒Teller (BET) surface area of P-Tri-RhO_2_ (30.3 m^2^ g^−1^) is 3.19 times larger than that of Rutile-RhO_2_ (9.5 m^2^ g^−1^) (Supplementary Fig. 5), indicating that P-Tri-RhO_2_ nanosheets may possess more surface area. The crystal lattice of the P-Tri-RhO_2_ sheet is clearly revealed in aberration-corrected dark-field scanning transmission electron microscopy (STEM-ADF) images (Fig. 1e–g), showing that one Rh atom is surrounded by six adjacent Rh atoms with an intersection angle of 60°. As the intensity of atomic columns is proportional to the atomic number, a hexagonal pattern of Rh atoms is found, while the intensity of oxygen columns is too weak to be seen. The distance between two adjacent Rh atoms is determined to be 0.31 nm via STEM-ADF imaging, which is almost the same as the XRD result. P-Tri-RhO_2_ completely transfers to rutile phase RhO_2_ (Rutile-RhO_2_) under annealing at 650 °C (Supplementary Fig. 6), reflecting its metastable nature.

In addition, the detailed electronic structures of P-Tri-RhO_2_ were studied by X-ray photoelectron spectroscopy (XPS) and synchrotron-based X-ray absorption spectroscopy (XAS). Information on the electronic state of P-Tri-RhO_2_ was first revealed by XPS (Supplementary Fig. 7). The peaks at 309.1 eV and 313.9 eV are completely attributed to Rh^4+^ 3*d*_5/2_ and Rh^4+^ 3*d*_3/2_ compared to those of Rutile-RhO_2_^38^, suggesting that a high purity phase of P-Tri-RhO_2_ is obtained by the radiofrequency-assisted molten-alkali method. X-ray absorption near-edge structure (XANES) and extended X-ray absorption fine structure (EXAFS) analyses are highly sensitive to the electronic structure and the local environment of transmission metal ions^39–42^. Figure 1h shows the Rh-K XANES spectra of P-Tri-RhO_2_ together with Rutile-RhO_2_ and Rh foil for comparison. The absorption edge of P-Tri-RhO_2_ is close to that of Rutile-RhO_2_, indicating that the valence state of Rh in P-Tri-RhO_2_ is close to +4. The Fourier transforms of P-Tri-RhO_2_, Rh foil and Rutile-RhO_2_^43^ in Fig. 1i show the scattering profile as a function of the radial distance from the central absorbing Rh atom. The first peak at approximately 1.7 Å is assigned to Rh-O coordination, as found in Rutile-RhO_2_^43^ (blue line) and P-Tri-RhO_2_ (black line), which have a second peak at approximately 2.8 Å related to the Rh-Rh shell. The spectrum for P-Tri-RhO_2_ is fitted, as shown in Supplementary Fig. 8a, with the two coordination shells corresponding to Rh-O and Rh-Rh shells. The coordination number and bond lengths for these two shells are fitted to 6/2.02 ± 0.16 Å and 6/3.10 ± 0.01 Å, respectively, which is consistent with the XRD and STEM-ADF results. All these results allow us to conclude that a phase of RhO_2_ with space group No. 164 (P-3m1) has been successfully prepared (Supplementary Table 1); the corresponding structure of P-Tri-RhO_2_ is clearly shown in Fig. 1j–l.

In general, metastable phase materials usually require harsh synthetic conditions because they have higher Gibbs free energies than thermodynamically stable phase materials. In our synthetic process, high energy is mainly supplied via radiofrequency heating due to its rapid heating capabilities, which contributes to the formation of metastable phase materials. Only the amorphous product is obtained when directly RhCl_3_ and KOH are directly mixed without the application of radiofrequency heating (Supplementary Fig. 9a, b). Rh_2_O_3_ is obtained when RhCl_3_ is directly heated without the addition of KOH (Supplementary Fig. 9c, d). The above experiments indicate the important roles of high energy input and alkaline conditions in synthesizing metastable phase materials. In addition, to verify the chemical stability of metastable P-Tri-RhO_2_, we performed more contrast experiments, as shown in Supplementary Fig. 10. The experimental results show that there are no morphology or crystal structure changes in P-Tri-RhO_2_ after different treatments, indicating its excellent chemical stability.

### Preparation and structural characterization of Rh-NA/RhO_2_

**Fig. 2:**
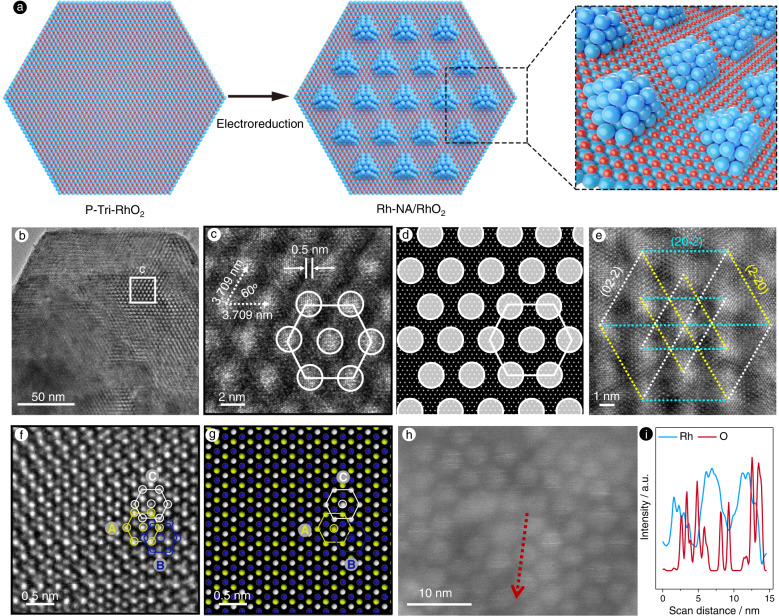
Structure characterization of Rh-NA/RhO_2_. **a** Schematic representation for in-situ growth of molecular-scale-spacing, well ordered and single crystalline Rh array on substrate of P-Tri-RhO_2_ to obtain Rh-NA/RhO_2_ electrocatalyst. **b** The STEM image of Rh-NA/RhO_2_ and (**c**) its partial enlargement, clearly showing single crystalline Rh atom array on the surface of P-Tri-RhO_2_. **d** Simulated pattern for (**c**). **e** The enlarged STEM images of Rh-NA/RhO_2_, clearly showing {220} crystal lattices of Rh particles with well ordered arrangement: (20-2), (2-20) and (02-2) planes were marked with the cyan, yellow and white colors respectively. **f** The further magnification for STEM image of Rh-NA/RhO_2_. **g** Simulated STEM image for (**f**), clearly showing three layers structure of face-centered cubic Rh. The Rh atoms on different layers are marked with green, red and white colours. **h** The HAADF-STEM image and (**i**) the corresponding EDX line scanning profile of Rh-NA/RhO_2_. Figure 2a was made with the Cinema 4D Software.

Well-ordered nanocrystal arrays (Rh-NA/RhO_2_) were then prepared by electrochemically reducing P-Tri-RhO_2_ by means of the chronoamperometry method at a constant reduction potential (vs. RHE) of −0.4 V for 2 h (Fig. 2a). The XRD pattern of Rh-NA/RhO_2_ suggests that only a small amount of metallic Rh formed in Rh-NA/RhO_2_ (Supplementary Fig. 11). In addition, the atomic ratio of metallic Rh and P-Tri-RhO_2_ in the Rh-NA/RhO_2_ electrocatalyst is approximately 1:4 according to the XPS results (Supplementary Fig. 12). Next, the electrochemical specific surface area (ECSAs) of Rh-NA/RhO_2_ was determined from hydrogen under potential deposition (ECSA_Hupd_) by obtaining cyclic voltammograms (CVs) in 0.5 M H_2_SO_4_ with a scan rate of 50 mV s^−1^. As shown in Supplementary Fig. 13 and Supplementary Table 2, the ECSA_Hupd_ of P-Tri-RhO_2_ increases from 8.8 to 55.5 m^2^ g_Rh_^−1^ after the 2 h electroreduction test, further suggesting the in situ formation of Rh nanoparticles on the P-Tri-RhO_2_ substrate.

To further demonstrate the structure of Rh-NA/RhO_2_, scanning transmission electron microscopy (STEM) images of Rh-NA/RhO_2_ are shown in Fig. 2b–d, which clearly reveal that the Rh nanocrystal array is uniformly distributed in a hexagonal pattern on the substrate of P-Tri-RhO_2_. A STEM image at high magnification shows that the Rh particles are single crystalline in nature with a diameter of ~3.2 nm. More importantly, the interparticle distance of this array is measured to be 3.709 nm, which means there is a molecular-scale distance (~0.5 nm) between two adjacent particles (Fig. 2c). As a comparison, we conducted the same in situ electrochemical reduction step for Rutile-RhO_2_, and the corresponding results are shown in Supplementary Fig. 14. The TEM and HRTEM images of the above product indicate that the Rh nanoparticles can be reduced in situ on Rutile-RhO_2_, while no Rh nanocrystal arrays are observed, suggesting the key role of the metastable two-dimensional P-Tri-RhO_2_ precursor in forming this special array structure (Supplementary Fig. 14b–d).

It is worth noting that these Rh single-crystal particles are well ordered, so all the crystal lattices of the Rh {220} planes are shown in the same orientation (Fig. 2e). The (20-2), (2-20) and (02-2) planes are marked in cyan, yellow and white, respectively. Such particles aligned in an arrayed manner may originate from the intrinsic anisotropy of metastable RhO_2_, which compels Rh particles to grow preferentially along certain crystallographic directions.

To better explain the growth mechanism for the single crystalline Rh array, the mismatch between the (20–20) plane of P-Tri-RhO_2_ and the (220) plane of face-centred cubic Rh is calculated, as shown in Supplementary Fig. 15. The cell parameters of *a* for P-Tri-RhO_2_ and face-centred cubic Rh are 0.3091 and 0.3803 nm, respectively. The values of the *d*-spacing of the P-Tri-RhO_2_ (20-20) plane and Rh (220) plane are determined to be 0.1339 and 0.1345 nm, respectively, indicating that there is only 0.45% ((0.1345–0.1339) × 100% / 0.1339 = 0.45%) mismatch. Such a small mismatch makes the in situ epitaxial growth of face-centred cubic Rh on the P-Tri-RhO_2_ substrate feasible. Further magnification of the STEM images of Rh-NA/RhO_2_ clearly proves that face-centred cubic Rh with a three-layer structure is generated in situ on the substrate of P-Tri-RhO_2_ by epitaxial growth along the [111] direction of Rh (Fig. 2f, g).

While these nanocrystal arrays are similar to Moire patterns at first glance, careful comparison shows that they are totally different. We first simulated Moire patterns by twisting the bilayer P-Tri-RhO_2_ region from 0 to 30° with increments of 1°, as shown in Supplementary Movie 1. These Moire patterns all have a similar arrangement. Taking the Moire pattern with a rotation angle of 3° as an example, a small region of the simulated Moire pattern (Supplementary Fig. 16a) is similar to the real experimental HRTEM image (Fig. 2c), but in larger regions, the Moire patterns (Supplementary Fig. 16b, c) are different from the real experimental and simulated patterns (Fig. 2f, g). Moreover, we compared the surface densities (SDs, the number of Rh atoms per nm^2^) of Rh atoms of Rh-NA/RhO_2_ and different Moire patterns. The theoretical maximum SDs of Rh atoms in the Moire patterns constructed by two single layers of P-Tri-RhO_2_, two single layers of metallic Rh, or a single layer of P-Tri-RhO_2_ and a single layer of metallic Rh are determined to be 24.172, 31.938 or 28.055 nm^−2^, respectively (Supplementary Note 1 and Supplementary Table 3), much lower than those of Rh nanocrystal arrays in Rh-NA/RhO_2_ (the theoretical and actual SDs are 47.904 and 47.3 ± 1.2 nm^−2^, respectively). We also simulated a Moire pattern by rotating a single layer of P-Tri-RhO_2_ and a single layer of metallic Rh. As shown in Supplementary Fig. 17, a typical Moire pattern is obtained by twisting a single layer of P-Tri-RhO_2_ and a single layer of metallic Rh with a rotation angle of 3°. The atomic enlarged areas of this Moire pattern are completely different from those of the Rh nanocrystal array (Fig. 2f, g), indicating that the Rh nanocrystal array is real rather than a Moire pattern. The Rh nanocrystal arrays are clearly observed in the HAADF-TEM image (Fig. 2h). The EDX line scanning profile in Fig. 2i shows that the contrasts on the Rh and O elements demonstrate nearly equal spaced Rh nanocrystals in a consistent way, which also excludes the formation of Moire pattern. Furthermore, we also simulated the XRD patterns of different atomic layers of Rh. As shown in Supplementary Fig. 18, the simulated XRD peaks of different atomic layers of Rh cannot be detected in Rh-NA/RhO_2_, excluding a thin Rh atomic layer on P-Tri-RhO_2_.

The XPS spectrum of Rh-NA/RhO_2_ is also shown in Supplementary Fig. 12, where the peaks at 307.3 eV and 312.1 eV are attributed to metallic Rh 3*d*_5/2_ and 3*d*_3/2_, respectively^44^. Compared with C-Rh/C, the binding energy of Rh in Rh-NA/RhO_2_ shifts to a higher binding energy by approximately 0.15 eV, indicating the existence of a strong electronic interaction between the Rh nanocrystal array and the P-Tri-RhO_2_ substrate^43^. As shown in Fig. 1h, the energetic position of Rh-NA/RhO_2_ is located between those of Rutile-RhO_2_ and the Rh foil, indicating that the Rh ions in Rh-NA/RhO_2_ are reduced^45,46^. As shown in Fig. 1i, for Rh-NA/RhO_2_, a peak occurs at 2.2 Å, which is located at the same position as that in Rh foil (green line), again indicating the reduction of Rh, in agreement with the Rh-K XANES results. The spectrum for Rh-NA/RhO_2_ is fitted and shown in Supplementary Fig. 8d with three obvious coordination shells corresponding to two shells for RhO_2_ and one for the reduced Rh. The coordination number and bonding lengths for these three shells are fitted to 6/3.10 ± 0.01 Å, 12/2.68 ± 0.08 Å and 6/2.02 ± 0.16 Å, respectively, clearly indicating that face-centred cubic Rh is generated in situ on the P-Tri-RhO_2_ substrate after electroreduction.

### Electrochemical performance of Rh-NA/RhO_2_

**Fig. 3:**
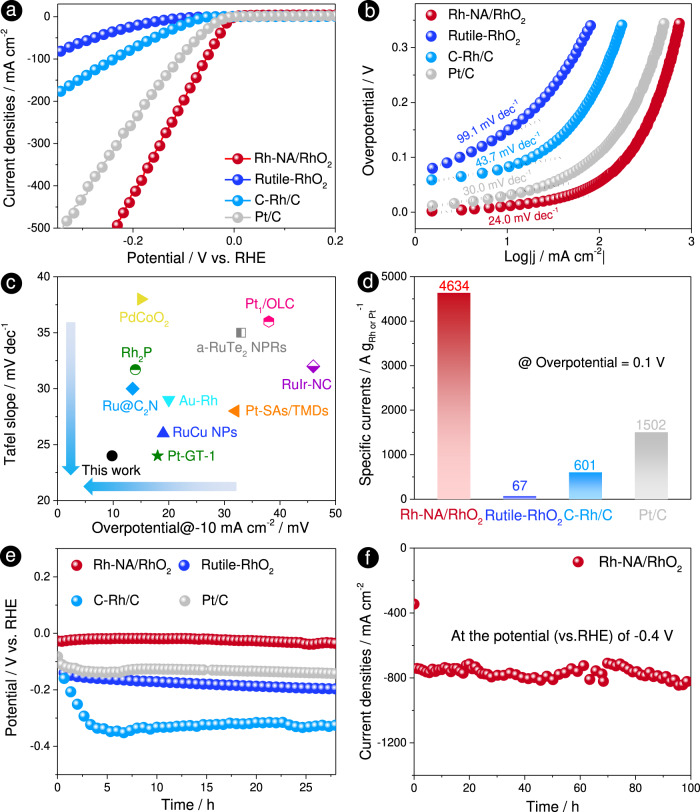
HER performances of Rh-NA/RhO_2_, Rutile-RhO_2_, C-Rh/C and Pt/C in H_2_-saturated 0.5 M H_2_SO_4_ electrolyte. **a** The HER polarization curves of Rh-NA/RhO_2_, Rutile-RhO_2_, C-Rh/C and Pt/C with *i*R-correction. **b** Tafel plots obtained from the polarization curves of Rh-NA/RhO_2_, Rutile-RhO_2_, C-Rh/C and Pt/C in Fig. 3a. **c** Comparison of Tafel slopes and overpotentials at the current density of −10 mA cm^−2^ for Rh-NA/RhO_2_ with previous reported high activity noble metal based HER catalysts (Pt-GT-1^59^, RuCu NPs^60^, Pt-SAs/TMDs^61^, Au-Rh^62^, Ru@C_2_N^63^, Rh_2_P^64^, RuIr-NC^65^, a-RuTe_2_ NPRs^66^, PdCoO_2_^67^ and Pt_1_/OLC^68^). **d** Comparison of specific currents for Rh-NA/RhO_2_, Rutile-RhO_2_, C-Rh/C and Pt/C electrocatalysts at the overpotential of 0.1 V. **e** Stability of Rh-NA/RhO_2_, Rutile-RhO_2_, C-Rh/C and Pt/C electrocatalysts by the chronopotentiometry technique at a constant current density of −10 mA cm^−2^. **f** The stability test for Rh-NA/RhO_2_ by chronoamperometry test under the high potential (vs. RHE) of −0.4 V for 100 h.

In the following study, we evaluated the HER catalytic activity of Rh-NA/RhO_2_ in the H_2_-saturated 0.5 M H_2_SO_4_ via a three-electrode system. Before electrochemical tests, two Pt electrodes were used as the working and counter electrodes to calibrate the SCE (Supplementary Fig. 19). The HER performances of Rh-NA/RhO_2_, Rutile-RhO_2_, C-Rh/C and Pt/C are shown in Fig. 3a, where Rh-NA/RhO_2_ exhibits better HER activity than Rutile-RhO_2_, C-Rh/C and Pt/C. The overpotentials at a current density of −10 mA cm^−2^ are summarized in Supplementary Fig. 20 and Supplementary Table 4, with which the HER activities can be quantitatively compared. In detail, Rh-NA/RhO_2_ delivers a low overpotential of 9.8 mV at a current density of −10 mA cm^−2^, which is much lower than those of Rutile-RhO_2_ (148 mV), C-Rh/C (83 mV) and Pt/C (29 mV). The corresponding Tafel slopes of Rh-NA/RhO_2_, Rutile-RhO_2_, C-Rh/C and Pt/C are 24.0, 99.1, 43.7 and 30.0 mV dec^−1^, respectively (Fig. 3b), indicating that Rh-NA/RhO_2_ exhibits the fastest kinetic rate towards hydrogen evolution.

The exchange current density (|j_0_|) of Rh-NA/RhO_2_ is 3.356 mA cm^−2^, 2.75 times higher than that of Pt/C (1.220 mA cm^−2^), indicating its excellent HER electrocatalytic activity (Supplementary Table 5). Then, the specific current of Rh-NA/RhO_2_ at −0.1 V (vs. RHE) is up to 4634 A g_Rh_^−1^, 69.1, 7.7 and 3.1 times higher than those of Rutile-RhO_2_ (67 A g_Rh_^−1^), C-Rh/C (601 A g_Rh_^−1^) and Pt/C (1502 A g_Pt_^−1^), respectively (Fig. 3d and Supplementary Fig. 21). The electrochemical specific surface areas form hydrogen under potential deposition (ECSA_Hupd_) of Rh-NA/RhO_2_, Rutile-RhO_2_, C-Rh/C and Pt/C are determined to be 55.5 m^2^ g_Rh_^−1^, 8.3 m^2^ g_Rh_^−1^, 15.3 m^2^ g_Rh_^−1^ and 47.3 m^2^ g_Pt_^−1^ respectively (Supplementary Fig. 22 and Supplementary Table 2). Next, the stability of Rh-NA/RhO_2_, Rutile-RhO_2_, C-Rh/C and Pt/C was evaluated by chronopotentiometry at a constant current density of −10 mA cm^−2^. In addition, the overpotential (vs. RHE) at −10 mA cm^−2^ and Tafel slope of Rh-NA/RhO_2_ are also compared with those of previously reported high-activity noble metal-based HER catalysts (Fig. 3c and Supplementary Table 6), showing that Rh-NA/RhO_2_ is among the best electrocatalysts for hydrogen evolution. To further eliminate the effect of particle size and surface area, the turnover frequencies (TOFs) were calculated to compare the catalytic activities of different catalysts. As shown in Supplementary Fig. 23a, Rh-NA/RhO_2_ always has a higher TOF than Rutile-RhO_2_, C-Rh/C and Pt/C at different potentials (vs. RHE). In detail, a TOF of 4.68 s^−1^ is achieved with Rh-NA/RhO_2_ at overpotentials of 20 mV, 17.3, 18 and 6.5 times higher than those of Rutile-RhO_2_ (0.27 s^−1^), C-Rh/C (0.26 s^−1^) and Pt/C (0.72 s^−1^) (Supplementary Fig. 23b), indicating the excellent HER intrinsic activity of Rh-NA/RhO_2_. As shown in Fig. 3e, the potential (vs. RHE) of Rh-NA/RhO_2_ shifts only 7 mV after the 28 h stability test. In a sharp comparison, the Rutile-RhO_2_, C-Rh/C and Pt/C drop 99, 166 and 60 mV after 28 h, respectively, suggesting that Rh-NA/RhO_2_ exhibits not only excellent HER activity but also long-term durability.

To reveal the structural change in the Rh nanocrystal array after the harsh long-term hydrogen evolution reaction, a chronoamperometry test was carried out under a high potential (vs. RHE) of −0.4 V. As shown in Fig. 3f, Rh-NA/RhO_2_ exhibits superior stability over a 100 h long-term test, with the average current density reaching −750 mA cm^−2^. The XRD pattern shows that there are no obvious crystal structure changes in Rh and Tri-RhO_2_ after the stability test (Supplementary Fig. 24a). SEM and STEM images of Rh-NA/RhO_2_ after the stability test indicate that the structural change in the Rh nanocrystal array is very limited (Supplementary Fig. 24b, c). XPS (C1*s*, O1*s* and F1*s*) and FTIR spectra of Rh-NA/RhO_2_ were collected after the long-term HER stability test and are shown in Supplementary Fig. 25. The C-F, CH_3_ and O-H bonds can be clearly observed from FTIR and XPS spectra, proving the existence of Nafion and isopropanol in the Rh-NA/RhO_2_ electrocatalyst^47,48^.

### The hydrogen spillover of Rh-NA/RhO_2_

The low overpotential and small Tafel slope of Rh-NA/RhO_2_ might be attributed to the unique interaction between the nanocrystal hexagonal array and the two-dimensional metastable substrate. The molecular-scale distance between two adjacent Rh particles is only 0.5 nm, favouring hydrogen spillover^49–51^. The functional properties of arrays heavily depend on their density, their interparticle distance, their orientation and the uniformity of the particles, as well as the crystal quality^52^. The arrays with large interparticle spacing maintain their individual properties, while those short interparticle distance exhibit strong coupling between particles^16^. As listed in Supplementary Table 7, R Rh-NA/RhO_2_ has the shortest interparticle distance, leading to strong coupling between adjacent particles in HER catalysis and thus exhibiting enhanced HER catalytic performance.

A hydrogen spillover-assisted HER mechanism for the Rh-NA/RhO_2_ catalyst was proposed, and a detailed process is shown in Supplementary Note 2. To confirm the hydrogen spillover phenomenon for the Rh-NA/RhO_2_ electrocatalyst, the physical mixtures of Rh-NA/RhO_2_ and WO_3_ were treated under a H_2_ atmosphere at room temperature (Supplementary Fig. 26b). As expected, the yellow WO_3_ particles turn dark blue^51^, indicating the existence of a hydrogen spillover effect in the Rh-NA/RhO_2_ electrocatalyst. We also performed the same experiments by using the physical mixtures of P-Tri-RhO_2_ and WO_3_ (P-Tri-RhO_2_-WO_3_) and Rh and WO_3_ (Rh-WO_3_). As shown in Supplementary Fig. 26c, no colour change is observed in P-Tri-RhO_2_-WO_3_, suggesting no hydrogen spillover generation. As shown in Supplementary Fig. 26d, the physical mixture of Rh-WO_3_ turns to a dark-blue colour after H_2_ treatment, indicating the existence of hydrogen spillover in metallic Rh.

We then performed experiments to reveal the active sites of Rh-NA/RhO_2_ for the hydrogen evolution process by adding thiocyanate (SCN^−^) or tetramethylammonium cation (TMA^+^) to an acidic electrolyte since SCN^−^ and TMA^+^ have specific interactions with metal and negative oxygenated species, respectively^53^. As shown in Supplementary Fig. 27, Rh-NA/RhO_2_ exhibits obvious performance decay after the addition of these two chemical probes, indicating that both Rh and RhO_2_ play important roles in the HER. However, the metallic Rh catalyst shows obvious performance loss after the addition of SCN^−^ and has nearly no performance decay after the addition of TMA^+^, showing that metallic Rh is the sole active site and that TMA^+^ has no effect on Rh.

### Theoretical simulation of the HER on Rh-NA/RhO_2_

**Fig. 4:**
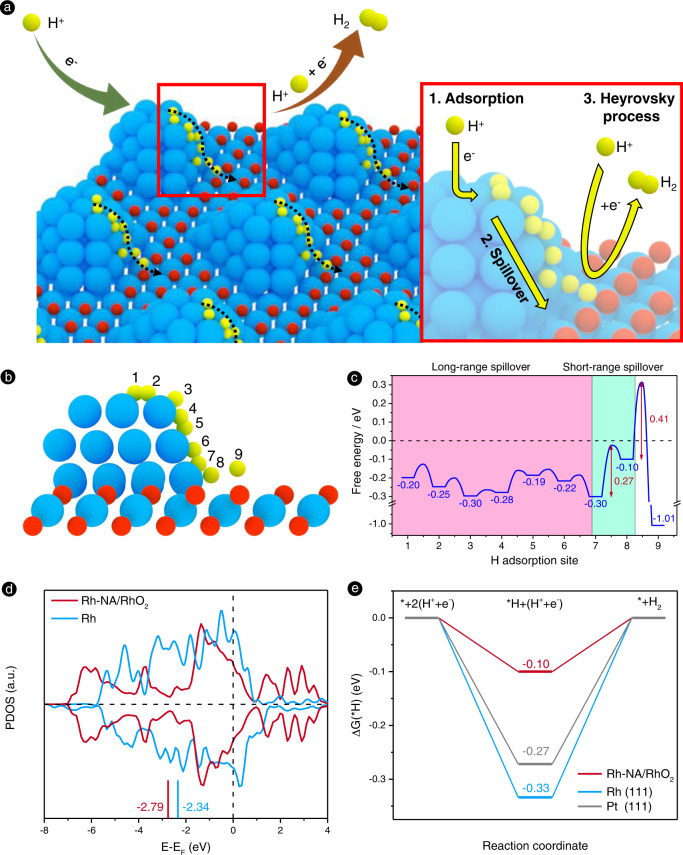
Schematic of hydrogen spillover assisted HER mechanism and DFT calculation of Gibbs free energy evolution. **a** The schematic representation of hydrogen spillover assisted HER mechanism for Rh-NA/RhO_2_ electrocatalyst. The enlarge image shows that the HER process in Rh-NA/RhO_2_ system includes three main steps: (i) adsorption and reduction of hydrogen on the Rh nanoparticles surface; (ii) the hydrogen spillover from Rh nanoparticles to the contact interface between Rh nanoparticles and P-Tri-RhO_2_ substrate; and (iii) the Heyrovsky process to produce hydrogen molecules. **b** The hydrogen spillover effect on Rh-NA/RhO_2_. **c** The hydrogen adsorption free energy diagram of different sites on Rh-NA/RhO_2_. **d** Comparisons of the *d*-orbitals distribution of Rh and Rh-NA/RhO_2_. **e** Two-electron Gibbs free energy evolution diagrams on Pt (111), Rh (111) and Rh-NA/RhO_2_.

Density functional theory (DFT) calculations were further employed to explore the underlying HER enhancement mechanism behind the unique interface structure of Rh-NA/RhO_2_. A Rh nanoparticle was placed on the basal plane of the (6 × 6) supercells of the 2D P-Tri-RhO_2_ nanosheet to represent the structural model of Rh-NA/RhO_2_. We calculated the hydrogen adsorption free energies ΔG(*H) on Rh-NA/RhO_2_ to investigate the potential HER active sites, including the Rh nanoparticle, the contact interface between the Rh nanoparticle and the P-Tri-RhO_2_ substrate, and the basal plane of the P-Tri-RhO_2_ substrate (Fig. 4a–c). It is found that the basal O of Tri-RhO_2_ (site 9) has a strong hydrogen affinity with a ΔG(*H) of −1.01 eV, making it hard for the *H to diffuse due to its high energy barrier, 1.32 eV. By comparison, the *H on Rh nanoparticles (sites 1~7) are weaker, with ΔG(*H) of −0.30~−0.19 eV. It should be noted that the ΔG(*H) on the contact interface (site 8) is −0.10 eV, which is closer to zero for an ideal HER activity favourable for H_2_ formation when combined with a free proton in solvation. Moreover, the largest energy barrier of hydrogen spillover is only 0.27 eV from site 7 to 8. Therefore, our DFT calculations confirm the long-range and short-range hydrogen migration from the Rh nanoparticle to the contact interface (from site 1–7 to site 8) for H_2_ formation. P-Tri-RhO_2_ acts as a charge collector, attracting electrons from Rh nanoparticles. Therefore, the *d*-band centre of Rh-NA/RhO_2_ decreases by −2.79 eV, which is lower than that of Rh (111) (−2.34 eV) (Fig. 4d and Supplementary Fig. 28). The decrease in the *d*-band centre is helpful for weakening the surface hydrogen adsorption and modulating ΔG(*H) from −0.33 eV on Rh(111) to −0.10 eV on Rh-NA/RhO_2_, closer to zero than the −0.27 eV on Pt(111) (Fig. 4e). This also explains why our synthesized Rh-NA/RhO_2_ exhibits better HER activity than Pt-based electrocatalysts in the experiments. Therefore, we ultimately can reveal the hydrogen spillover-enhanced HER process in the Rh-NA/RhO_2_ system (Fig. 4a): (i) adsorption and reduction of hydrogen on Rh nanoparticles; (ii) hydrogen spillover from Rh nanoparticles to the contact interface; and (iii) the Heyrovsky process to produce hydrogen molecules on the contact interface.

## Discussion

In conclusion, a metastable oxide, trigonal phase RhO_2_, was successfully obtained via a radiofrequency heating method. Using trigonal phase RhO_2_ as the precursor, a well-ordered nanocrystal array on trigonal rhodium oxide was formed with a limited intersurface distance of 0.5 nm. The unique coupling between the nanocrystal array and the 2D metastable substrate enables effective hydrogen spillover, enhancing the hydrogen evolution reaction, with an ultralow Tafel slope of 24.0 mV dec^−1^ and an overpotential of only 9.8 mV at a current density of −10 mA cm^−2^. This work yields an important method for fabricating a well-ordered nanocrystal array with subnanometre spacing for future advanced applications.

## Methods

### Chemicals

Rhodium (III) chloride (RhCl_3_) was purchased from Aladdin Industrial Co. 10% Rh/C was purchased from Shanghai Haohong Biomedical Technology Co. 20% Pt/C was obtained from Aladdin Industrial Co. Potassium hydroxide and potassium thiocyanate (KOH, 99%) were purchased from Sinopharm Chemical Reagent Co. Hydrochloric acid (HCl, Guaranteed reagent) was purchased by Chinasun Specialty Products Co. Nafion solution (5 wt%) and Tetramethylammonium chloride were supported by Sigma–Alddrich Co. Isopropanol (99.8%) was obtained from Sinopharm Chemical Reagent Co. Toray carbon paper (TGP-H-60) was bought from Alfa Aesar. Other reagents were of analytical reagent grade without further purification. Double-distilled water was used all experiments.

### Synthesis of P-Tri-RhO_2_

The pristine trigonal RhO_2_ (P-Tri-RhO_2_) was obtained via a radio-frequency heating method. Specially, 300 mg RhCl_3_ and 10 g KOH were mixed in a high-quality corundum crucible. Then the radio frequency heater with the power of 15 kW was used to heat above mixture for 20 mins, and then naturally cooled to room temperature. The obtained product was washed by 1 M HCl and redistilled water for several times respectively, dried in air for 6 h to obtain P-Tri-RhO_2_. Rutile-RhO_2_ was obtained by annealing P-Tri-RhO_2_ at 650 °C in air for 2 h.

### Synthesis of Rh-NA/RhO_2_

Rh nanocrystal array (Rh-NA/RhO_2_) was obtained by in-situ growth of face-centered cubic Rh nanoparticles on the surface of P-Tri-RhO_2_ via the electroreduction method. In brief, 20 mg P-Tri-RhO_2_ was added into the mixed solution (9 mL isopropanol and 1 mL 0.5 wt% Nafion solution) and ultrasonicated to form the homogenous ink. 2 mL above dispersion was dropped on the surface of carbon paper (1 cm × 2 cm) and dried naturally. Then P-Tri-RhO_2_ was reduced by conducting chronoamperometry method at a constant potential (vs. RHE) of −0.4 V for 2 h. After that, the above product was collected from carbon paper, cleaned by ethanol for several times and dried in air for 6 h to obtain the final product of Rh-NA/RhO_2_.

### Structure characterization

X-ray powder diffraction (XRD, Philips X’pert PRO MPD diffractometer) with Cu Kα radiation source (λ_Cu_ = 0.15406 nm) was applied to study the phase and crystallography of all samples. The transmission electron microscopy (TEM) images and energy dispersive X-ray spectroscopy (EDX) of all samples were characterized via a FEI Tecnai F20 transmission electron microscope with an accelerating voltage of 200 kV. The content of O element in P-Tri-RhO_2_ was determined by elemental analysis method (elementar EL III). Scanning transmission electron microscopy (STEM) results were collected on a fifth order aberration-corrected transmission electron microscope (JEOL ARM200CF) at 80 kV. The images have been filtered using a Gaussian filter to improve the contrast. Samples were baked at 140 °C for 8 h before taking into the microscope. Scanning electron microscopy (SEM) was performed by using a Zeiss G500. The chemical states of products were analyzed by using X-ray photoelectron spectroscopy (XPS) on a Kratos AXIS UltraDLD ultrahigh vacuum surface analysis system with Al Kα radiation (1486 eV) as a probe. The surface topographic height of P-Tri-RhO_2_ was measured via the atomic force microscopy (AFM, Bruker Dimension Icon). The BET specific surface areas were characterized by American Micromeritics ASAP-2020 porosimeter. XAS data were collected at the SPring-8 BL14B2 (Harima Science Garden City, Hyogo) using a Si (111) quick-scanning monochromator with the transmission mode.

### Electrochemical measurements

CHI 760D electrochemical workstation with a standard three-electrode system was used to perform the HER experiments. A modified glassy carbon electrode (GCE, 3 mm in diameter or 0.0707 cm^−2^ in area) and a saturated calomel electrode (SCE) were chosen as the working electrode and the reference electrode, respectively. A carbon rod was selected as the counter electrode. The catalysts solution was prepared as follows: 1 mg catalyst (Rh-NA/RhO_2_ or Rutile-RhO_2_) and 4 mg carbon black were added into the mixed solution (900 μL isopropanol and 100 μL 0.5 wt% Nafion solution) and ultrasonicated to form the homogenous ink. 4 μL (20 μg catalyst or 3.1 μg Rh) dispersion was dropped on the surface of GCE (43.8 μg cm_Rh_^−2^) and dried naturally for testing. In this work, isopropanol is served as a dispersant agent. Nafion is served as dispersant and binding ones and affects the proton transfer. Both of them are important for the dispersity, stability and proton conduction of the electrocatalysts during the HER process. In addition, 5 mg 20% Pt/C or 10% Rh/C electrocatalysts were prepared as same as above method. Then 4 μL (4 μg Pt or 2 μg Rh) dispersion was dropped on the surface of GCE (56.5 μg cm_Pt_^−2^ or 28.3 μg cm_Rh_^−2^) and dried naturally for testing. The HER performances were analyzed in H_2_-saturated 0.5 M H_2_SO_4_ by linear sweep voltammetry (LSV) in the range of −0.4 to 0.2 V (vs. RHE) with the scan rate of 5 mV s^−1^ and 95% iR-compensation. The electrochemical specific surface areas (ECSAs) of different catalysts determined from hydrogen under potential deposition by performing CV in 0.5 M H_2_SO_4_ with a scan rate of 50 mV s^−1^ and ranging from 0 to 1.2 V (vs. RHE). The stability tests of Rh-NA/RhO_2_, Rutile-RhO_2_, C-Rh/C and Pt/C electrocatalysts by the chronopotentiometry technique were carried out at a constant current density of −10 mA cm^−2^. The stability test for Rh-NA/RhO_2_ by chronoamperometry test was carried out under the high potential (vs. RHE) of −0.4 V for 100 h. All electrochemical tests were carried out in ambient condition.

The corresponding equations are shown as follow:

Turnover frequencies (TOFs) of electrocatalysts were calculated as follows:

1 $${TOF}=\frac{3.12\times 1{0}^{15}\frac{{H}_{2}/s}{c{m}^{2}}{per}\frac{{mA}}{c{m}^{2}}\times {{{{{\rm{|}}}}}}j{{{{{\rm{|}}}}}}}{{active}\,{sites}}$$

The active sites of electrocatalysts was calculated as follows:

2 $${Active}\,{sites}=\frac{Q}{1.602\times 1{0}^{-19}}$$

3 $$Q=\frac{{S}_{{peak}}}{v}$$

Where S_peak_ is the integral area of adsorbed hydrogen desorption peak in the CV curve (Supplementary Fig. 22), v is the scan rate of 50 mV s^−1^ and Q is quantity of electric charge.

### Details of theoretical simulation

All theoretical simulations were under the framework of density functional theory with spin-polarized plane wave basis sets and implemented in the Vienna ab-initio Software Package (VASP) version 5.4.1^54,55^. The electronic exchange-correlation energy was described by the Perdew-Burke-Ernzerhof functional formula with consideration of the DFT-D3 correction method^56,57^. As the evaluation of electronic energies during self-consistent calculations, the cutoff energy was set to 450 eV and the convergence thresholds of energy and force were corresponding to 1E^−4^ eV and −0.05 eV Å^−1^, respectively. The k-point sampling only adopted Gamma point, which is enough to simulate this large metal-support model. The hydrogen adsorption free energy was calculated by

4 $$\varDelta {{{{{\rm{G}}}}}}(\ast {{{{{\rm{H}}}}}})=\varDelta {{{{{\rm{E}}}}}}+0.29={{{{{\rm{E}}}}}}(\ast {{{{{\rm{H}}}}}})-{{{{{\rm{E}}}}}}(\ast )-{{{{{\rm{E}}}}}}({{{{{{\rm{H}}}}}}}_{2})+0.29$$

in which the E(*H) and E(*) are the total energies of surface models with and without hydrogen adsorption, and the E(H_2_) is the total energy of hydrogen molecule. The constant of 0.29 is regarded as the contribution of vibrational and entropic correction^58^.

## Supplementary information

Supplementary Information

Peer Review File

Description of Additional Supplementary Files

Supplementary Movie 1

## Data Availability

The data generated in this study are provided in the Supplementary Information/Source Data file. Source data are provided with this paper.

## Source data

Source Data

## References

[1] Seh ZW (2017). Combining theory and experiment in electrocatalysis: insights into materials design. Science.

[2] Boles MA, Engel M, Talapin DV (2016). Self-assembly of colloidal nanocrystals: from intricate structures to functional materials. Chem. Rev..

[3] van der Hoeven JES (2021). Unlocking synergy in bimetallic catalysts by core-shell design. Nat. Mater..

[4] Fang S (2020). Uncovering near-free platinum single-atom dynamics during electrochemical hydrogen evolution reaction. Nat. Commun..

[5] Zhu JQ (2019). Boundary activated hydrogen evolution reaction on monolayer MoS_2_. Nat. Commun..

[6] Jiang H (2019). Defect-rich and ultrathin N doped carbon nanosheets as advanced trifunctional metal-free electrocatalysts for ORR, OER and HER. Energy Environ. Sci..

[7] Quaino P, Juarez F, Santos E, Schmickler W (2014). Volcano plots in hydrogen electrocatalysis-uses and abuses. Beilstein J. Nanotechnol..

[8] Costentin C, Saveant JM (2017). Towards an intelligent design of molecular electrocatalysts. Nat. Rev. Chem..

[9] Zhu LL (2016). A rhodium/silicon co-electrocatalyst design concept to surpass platinum hydrogen evolution activity at high overpotentials. Nat. Commun..

[10] Novoselov KS (2004). Electric field effect in atomically thin carbon films. Science.

[11] Li Y, Duan GT, Liu GQ, Cai WP (2013). Physical processes-aided periodic micro/nanostructured arrays by colloidal template technique: fabrication and applications. Chem. Soc. Rev..

[12] Barrigon E, Heurlin M, Bi ZX, Monemar B, Samuelson L (2019). Synthesis and applications of III-V nanowires. Chem. Rev..

[13] Kostiainen MA (2013). Electrostatic assembly of binary nanoparticle superlattices using protein cages. Nat. Nanotechnol..

[14] Huang N, Wang P, Jiang DL (2016). Covalent organic frameworks: A materials platform for structural and functional designs. Nat. Rev. Mater..

[15] Sofos M (2009). A synergistic assembly of nanoscale lamellar photoconductor hybrids. Nat. Mater..

[16] Nozik AJ (2010). Semiconductor quantum dots and quantum dot arrays and applications of multiple exciton generation to third-generation photovoltaic solar cells. Chem. Rev..

[17] Henry CR (2015). 2D-Arrays of nanoparticles as model catalysts. Catal. Lett..

[18] Motagamwala AH, Dumesic JA (2021). Microkinetic modeling: a tool for rational catalyst design. Chem. Rev..

[19] Nguyen PV (2019). Visualizing electrostatic gating effects in two-dimensional heterostructures. Nature.

[20] Chung YJ (2021). Ultra-high-quality two-dimensional electron systems. Nat. Mater..

[21] Schedin F (2007). Detection of individual gas molecules adsorbed on graphene. Nat. Mater..

[22] Akinwande D (2019). Graphene and two-dimensional materials for silicon technology. Nature.

[23] Liu J (2015). Metal-free efficient photocatalyst for stable visible water splitting *via* a two-electron pathway. Science.

[24] Liu CJ (2021). Two-dimensional superconductivity and anisotropic transport at KTaO_3_ (111) interfaces. Science.

[25] Kang MG (2018). Holstein polaron in a valley-degenerate two-dimensional semiconductor. Nat. Mater..

[26] Bergeron H, Lebedev D, Hersam MC (2021). Polymorphism in post-dichalcogenide two-dimensional materials. Chem. Rev..

[27] Jaramillo TF (2007). Identification of active edge sites for electrochemical H_2_ evolution from MoS_2_ nanocatalysts. Science.

[28] Chia X, Pumera M (2018). Characteristics and performance of two-dimensional materials for electrocatalysis. Nat. Catal..

[29] Peto J (2018). Spontaneous doping of the basal plane of MoS_2_ single layers through oxygen substitution under ambient conditions. Nat. Chem..

[30] Jin HY (2018). Emerging two-dimensional nanomaterials for electrocatalysis. Chem. Rev..

[31] Fan FR, Wang RX, Zhang H, Wu WZ (2021). Emerging beyond-graphene elemental 2D materials for energy and catalysis applications. Chem. Soc. Rev..

[32] Tan CL (2017). Recent advances in ultrathin two-dimensional nanomaterials. Chem. Rev..

[33] Chen Y (2020). Phase engineering of nanomaterials. Nat. Rev. Chem..

[34] Zavabeti A (2017). A liquid metal reaction environment for the room-temperature synthesis of atomically thin metal oxides. Science.

[35] Zhang BY (2021). Hexagonal metal oxide monolayers derived from the metal-gas interface. Nat. Mater..

[36] Li JY (2021). A fundamental viewpoint on the hydrogen spillover phenomenon of electrocatalytic hydrogen evolution. Nat. Commun..

[37] Dai J (2022). Hydrogen spillover in complex oxide multifunctional sites improves acidic hydrogen evolution electrocatalysis. Nat. Commun..

[38] Chen W, Wang H, Mao LQ, Chen XP, Shangguan WF (2014). Influence of loading Pt, RhO_2_ co-catalysts on photocatalytic overall water splitting over H_1.9_K_0.3_La_0.5_Bi_0.1_Ta_2_O_7_. Catal. Commun..

[39] Zhu T (2020). High-index faceted RuCo nanoscrews for water electrosplitting. Adv. Energy Mater..

[40] Agrestini S (2019). Nature of the magnetism of iridium in the double perovskite Sr_2_CoIrO_6_. Phys. Rev. B.

[41] Wu XH (2021). Fast operando spectroscopy tracking in situ generation of rich defects in silver nanocrystals for highly selective electrochemical CO_2_ reduction. Nat. Commun..

[42] Liu HJ (2017). Insight into the role of metal-oxygen bond and O 2p hole in high-voltage cathode LiNi_x_Mn_2-x_O_4_. J. Phys. Chem. C..

[43] Li Z (2020). Stable rhodium (IV) oxide for alkaline hydrogen evolution reaction. Adv. Mater..

[44] Meng XY (2020). Distance synergy of MoS_2_-confined rhodium atoms for highly efficient hydrogen evolution. Angew. Chem. Int. Ed..

[45] Liu S (2021). Ultrafine noble metal nanoclusters for unexpected anodic electrocatalysis. Chem. Catal..

[46] Zhang N (2020). Surface-regulated rhodium-antimony nanorods for nitrogen fixation. Angew. Chem. Int. Ed..

[47] Kosseoglou D, Kokkinofta R, Sazou D (2011). FTIR spectroscopic characterization of NafionA (R)-polyaniline composite films employed for the corrosion control of stainless steel. J. Solid. State Electr..

[48] del Arco M, Gutierrez S, Martin C, Rives V (2001). FTIR study of isopropanol reactivity on calcined layered double hydroxides. Phys. Chem. Chem. Phys..

[49] Karim W (2017). Catalyst support effects on hydrogen spillover. Nature.

[50] Savva PG, Efstathiou AM (2008). The influence of reaction temperature on the chemical structure and surface concentration of active NO_x_ in H_2_-SCR over Pt/MgO-CeO_2_: SSITKA-DRIFTS and transient mass spectrometry studies. J. Catal..

[51] Jiang LZ (2020). Facet engineering accelerates spillover hydrogenation on highly diluted metal nanocatalysts. Nat. Nanotechnol..

[52] Kim W, Guniat L, Morral AFI, Piazza V (2021). Doping challenges and pathways to industrial scalability of III-V nanowire arrays. Appl. Phys. Rev..

[53] Huang ZF (2019). Chemical and structural origin of lattice oxygen oxidation in Co-Zn oxyhydroxide oxygen evolution electrocatalysts. Nat. Energy.

[54] Kresse G, Furthmuller J (1996). Efficient iterative schemes for ab initio total-energy calculations using a plane-wave basis set. Phys. Rev. B.

[55] Kresse G, Furthmuller J (1996). Efficiency of ab-initio total energy calculations for metals and semiconductors using a plane-wave basis set. Comput. Mater. Sci..

[56] Perdew JP, Burke K, Ernzerhof M (1996). Generalized gradient approximation made simple. Phys. Rev. Lett..

[57] Grimme S, Antony J, Ehrlich S, Krieg H (2010). A consistent and accurate ab initio parametrization of density functional dispersion correction (DFT-D) for the 94 elements H-Pu. J. Chem. Phys..

[58] Choi WI, Wood BC, Schwegler E, Ogitsu T (2015). Combinatorial search for high-activity hydrogen catalysts based on transition-metal-embedded graphitic carbons. Adv. Energy Mater..

[59] Tiwari JN (2018). Multicomponent electrocatalyst with ultralow Pt loading and high hydrogen evolution activity. Nat. Energy.

[60] Yao Q (2019). Channel-rich RuCu nanosheets for pH-universal overall water splitting electrocatalysis. Angew. Chem. Int. Ed..

[61] Shi Y (2021). Electronic metal-support interaction modulates single-atom platinum catalysis for hydrogen evolution reaction. Nat. Commun..

[62] Du R (2020). Engineering multimetallic aerogels for pH-universal HER and ORR electrocatalysis. Adv. Energy Mater..

[63] Mahmood J (2017). An efficient and pH-universal ruthenium-based catalyst for the hydrogen evolution reaction. Nat. Nanotechnol..

[64] Yang FL (2018). A monodisperse Rh_2_P-based electrocatalyst for highly efficient and pH-universal hydrogen evolution reaction. Adv. Energy Mater..

[65] Wu DS (2021). Efficient overall water splitting in acid with anisotropic metal nanosheets. Nat. Commun..

[66] Wang J (2019). Amorphization activated ruthenium-tellurium nanorods for efficient water splitting. Nat. Commun..

[67] Podjaski F (2020). Rational strain engineering in delafossite oxides for highly efficient hydrogen evolution catalysis in acidic media. Nat. Catal..

[68] Li DB (2019). Atomically dispersed platinum supported on curved carbon supports for efficient electrocatalytic hydrogen evolution. Nat. Energy.

