# Supplementary material for: Coupling of nanocrystal hexagonal array and two-dimensional metastable substrate boosts H2-production
Source: Nat Commun. 2022 Oct 3;13:5828. doi: 10.1038/s41467-022-33512-5 (PMC9530234; doi:10.1038/s41467-022-33512-5)
Supplement: Supplementary file 1 — Supplementary Information [file 41467_2022_33512_MOESM1_ESM.pdf]

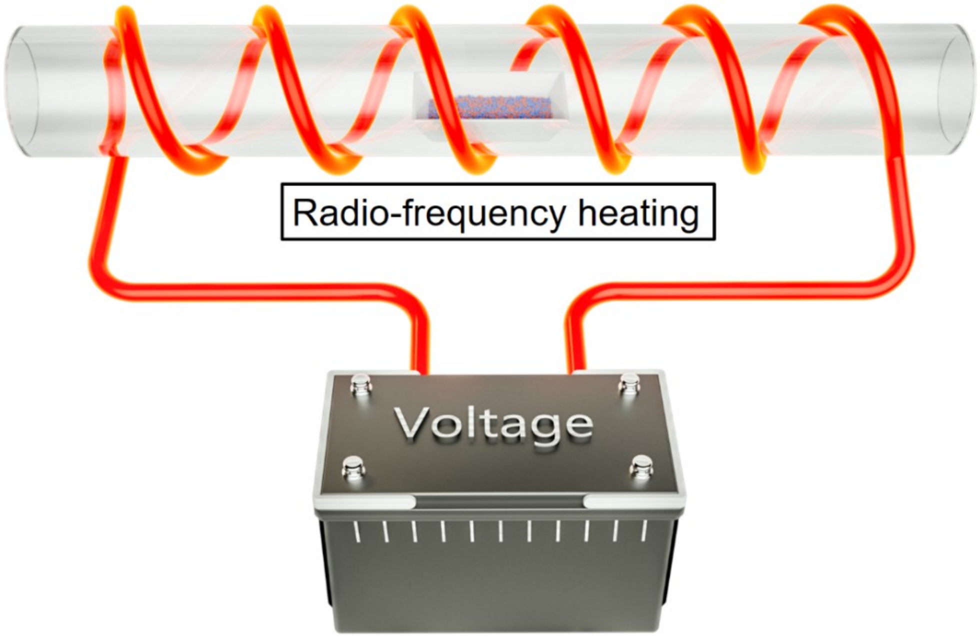

Supplementary Figure 1. Schematic representation of radio-frequency assisted molten-alkali method. The rhodium (III) chloride ($RhCl_3$) and potassium hydroxide (KOH) were selected as the raw materials and uniformly mixed in crucible. This figure was made with the Cinema 4D Software.

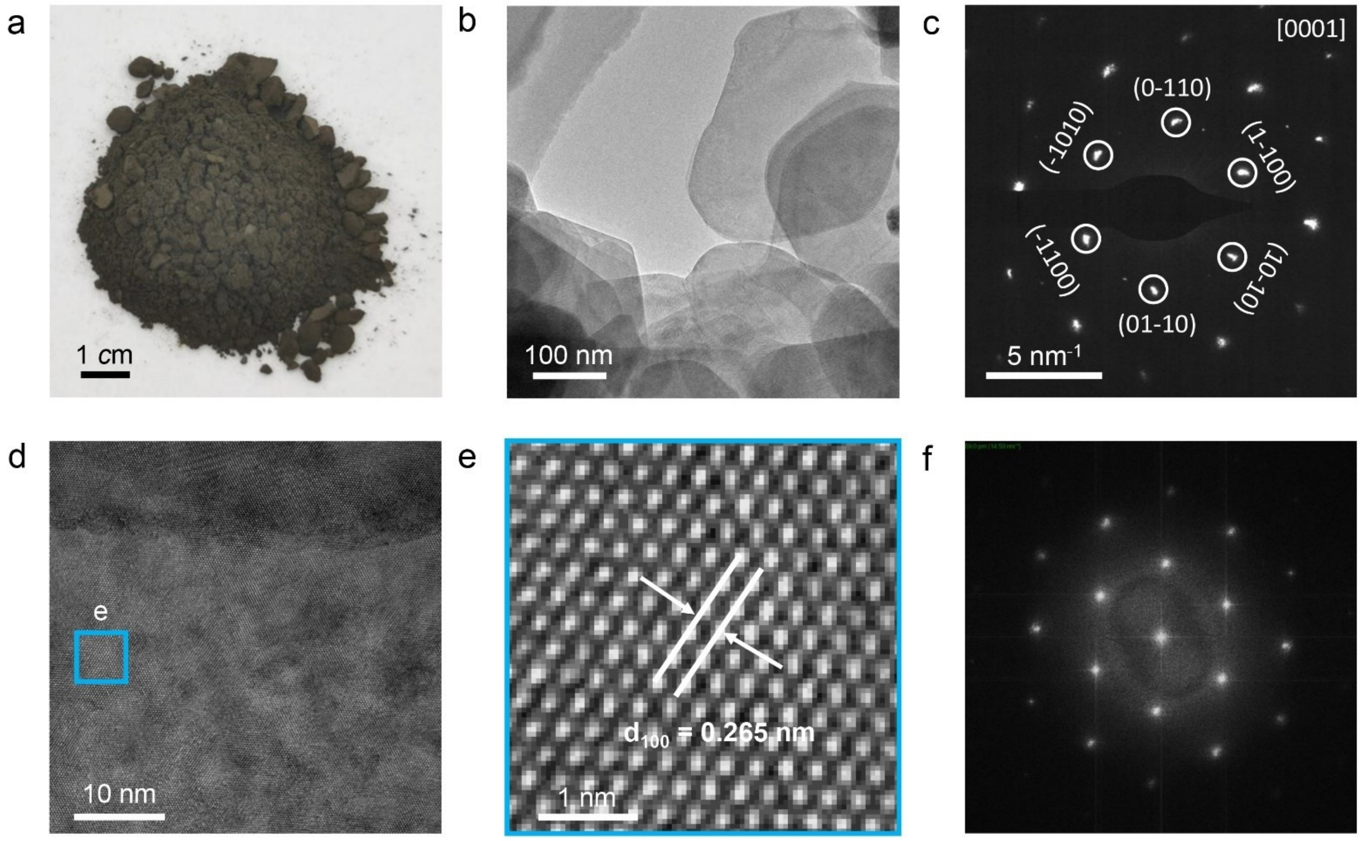

Supplementary Figure 2. Digital image, SEM, SAED, HRTEM and FFT images of P-Tri-RhO_2. (**a**) The digital image of P-Tri-RhO_2 powder. (**b**) TEM image and (**c**) SAED pattern of P-Tri-RhO_2. (**d**) HRTEM image and (**e**) its partially enlarged view. (**f**) The fast Fourier transform (FFT) from (**e**).

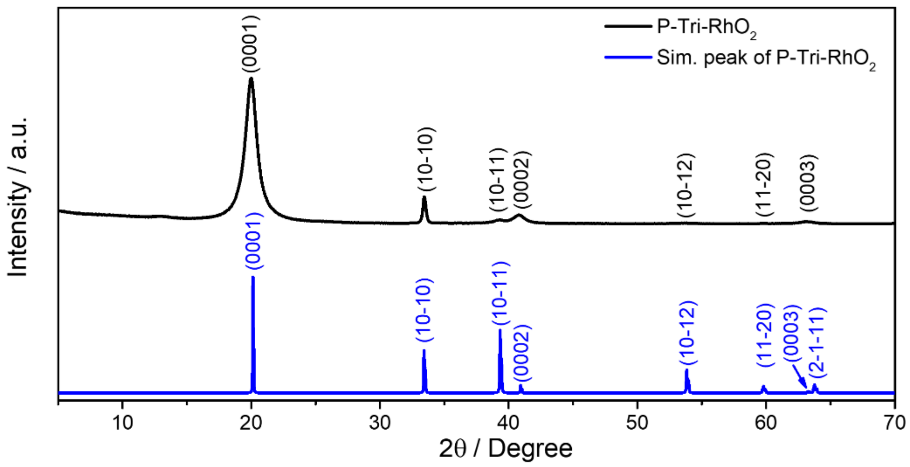

Supplementary Figure 3. Comparison of XRD pattern and simulated XRD pattern for P-Tri-RhO_2. Comparison of XRD patterns of P-Tri-RhO_2 (black curve) and simulated X-ray diffraction peak of P-Tri-RhO_2 (red curve).

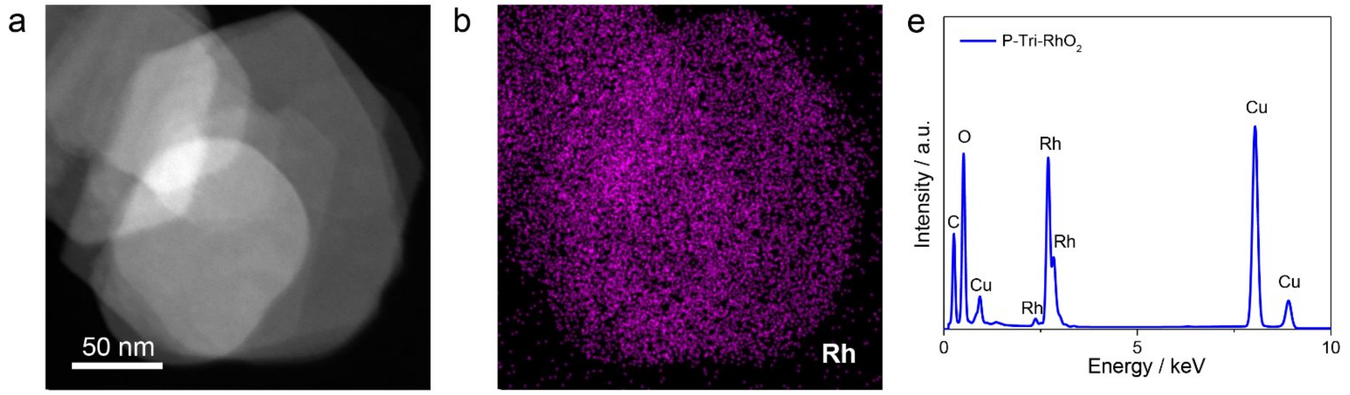

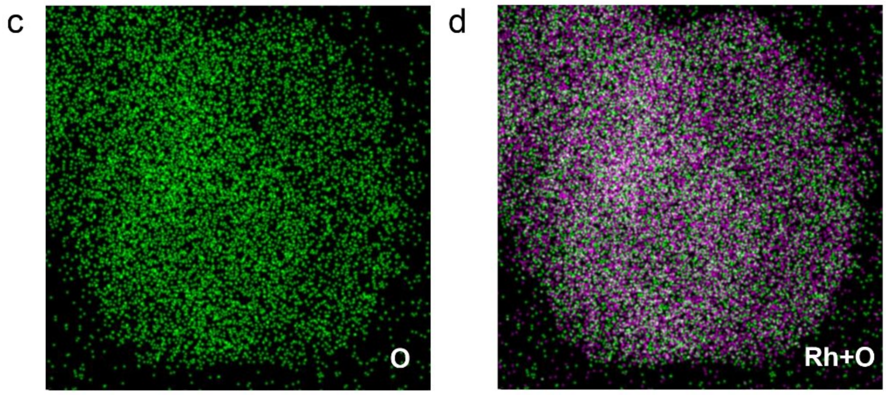

f

Element (Mass %)	Rh	O
P-Tri-RhO_2	76.7	23.3
**Element (Atomic %)**	**Rh**	**O**
P-Tri-RhO_2	33.8	66.2

Supplementary Figure 4. **EDX and elemental analysis of P-Tri-RhO_2.** (**a-d**) EDX-mapping images of P-Tri-RhO_2, where Rh and O are uniformly distributed in the nanosheet structure. (**e**) EDX analysis of P-Tri-RhO_2. (**f**) The table of the mass and atomic ratios of Rh to O in P-Tri-RhO_2 by elemental analysis method (elementar EL III).

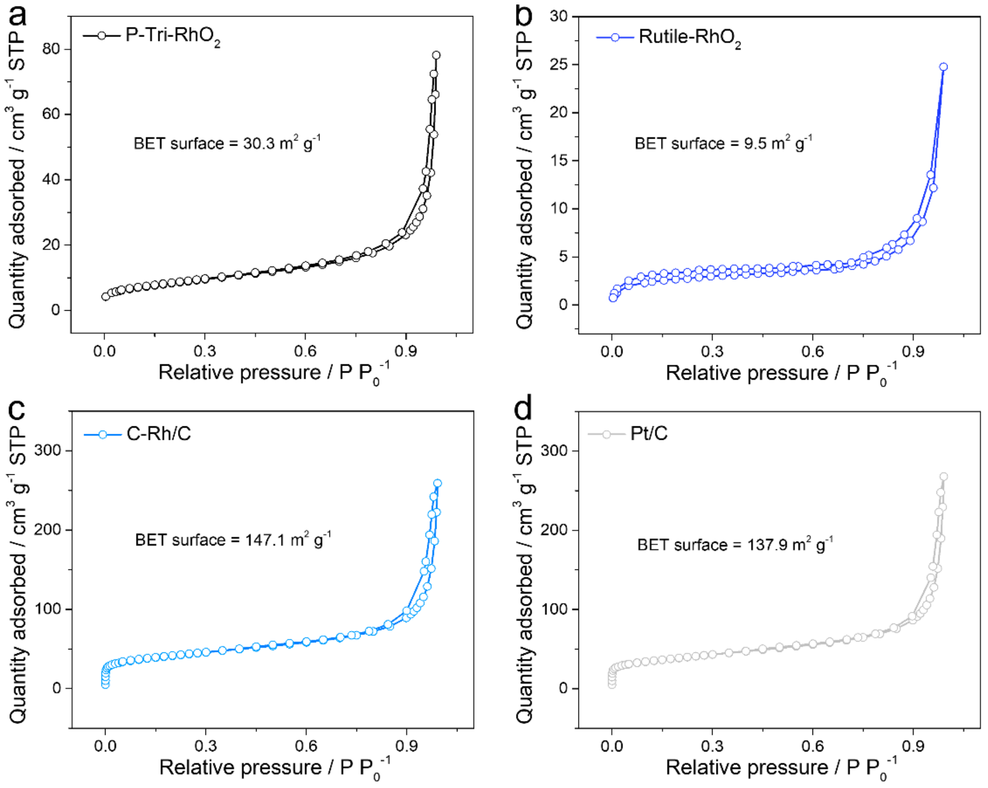

Supplementary Figure 5. BET surface area measurements for different catalysts. BET surface area measurements for (**a**) P-Tri-RhO_2, (**b**) Rutile-RhO_2, (**c**) C-Rh/C and (**d**) Pt/C.

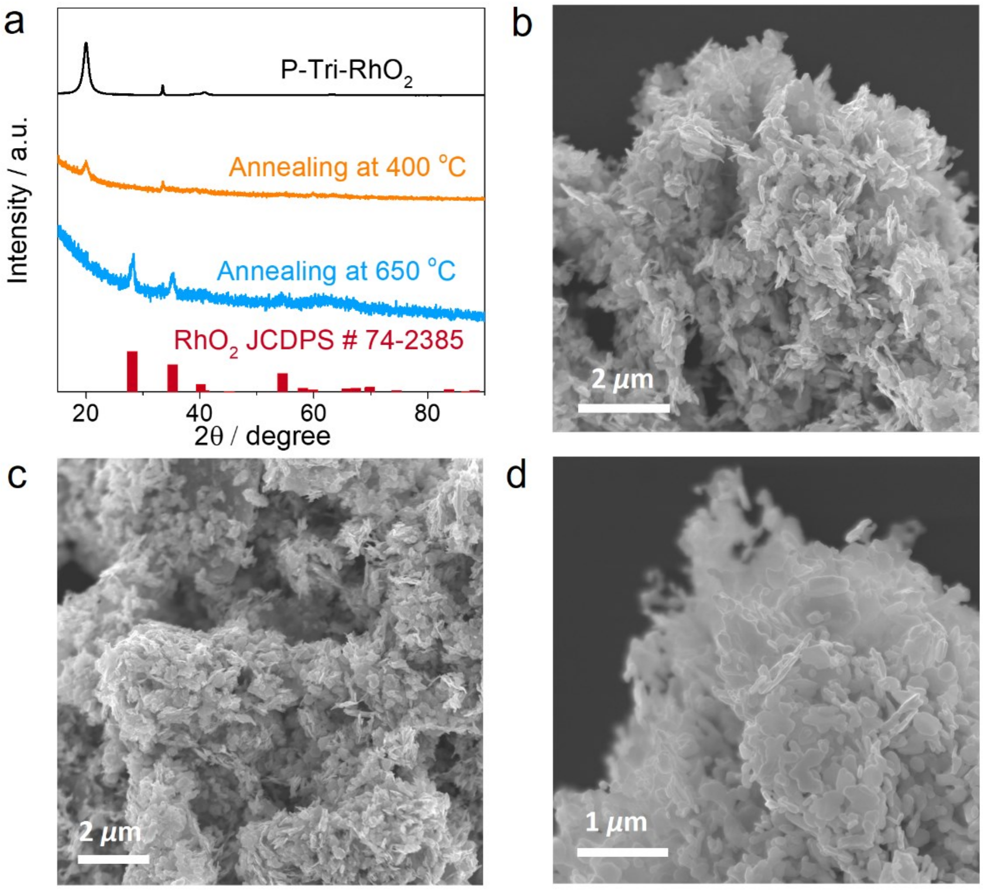

Supplementary Figure 6. XRD patterns and SEM images obtained by annealing P-Tri-RhO_2 at different temperatures. (**a**) Temperature-dependent XRD patterns of annealing P-Tri-RhO_2 at different temperatures. The corresponding SEM images of (**b**) P-Tri-RhO_2 and the products by annealing P-Tri-RhO_2 at (**c**) 400 °C and (**d**) 650 °C.

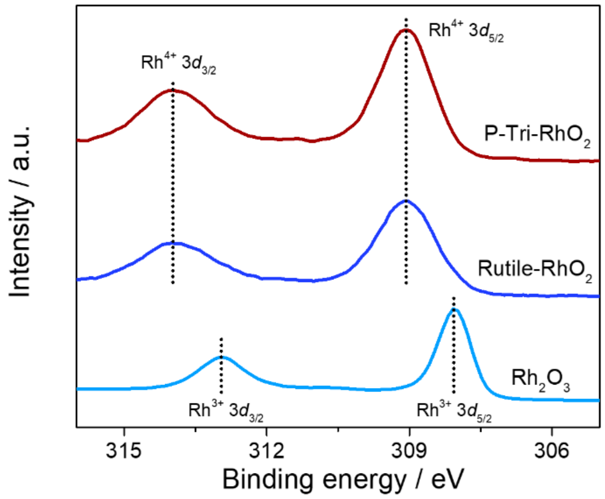

Supplementary Figure 7. XPS measurements for different samples. XPS spectra of Rh 3*d* peaks for P-Tri-RhO_2, Rutile-RhO_2 and Rh_2O_3.

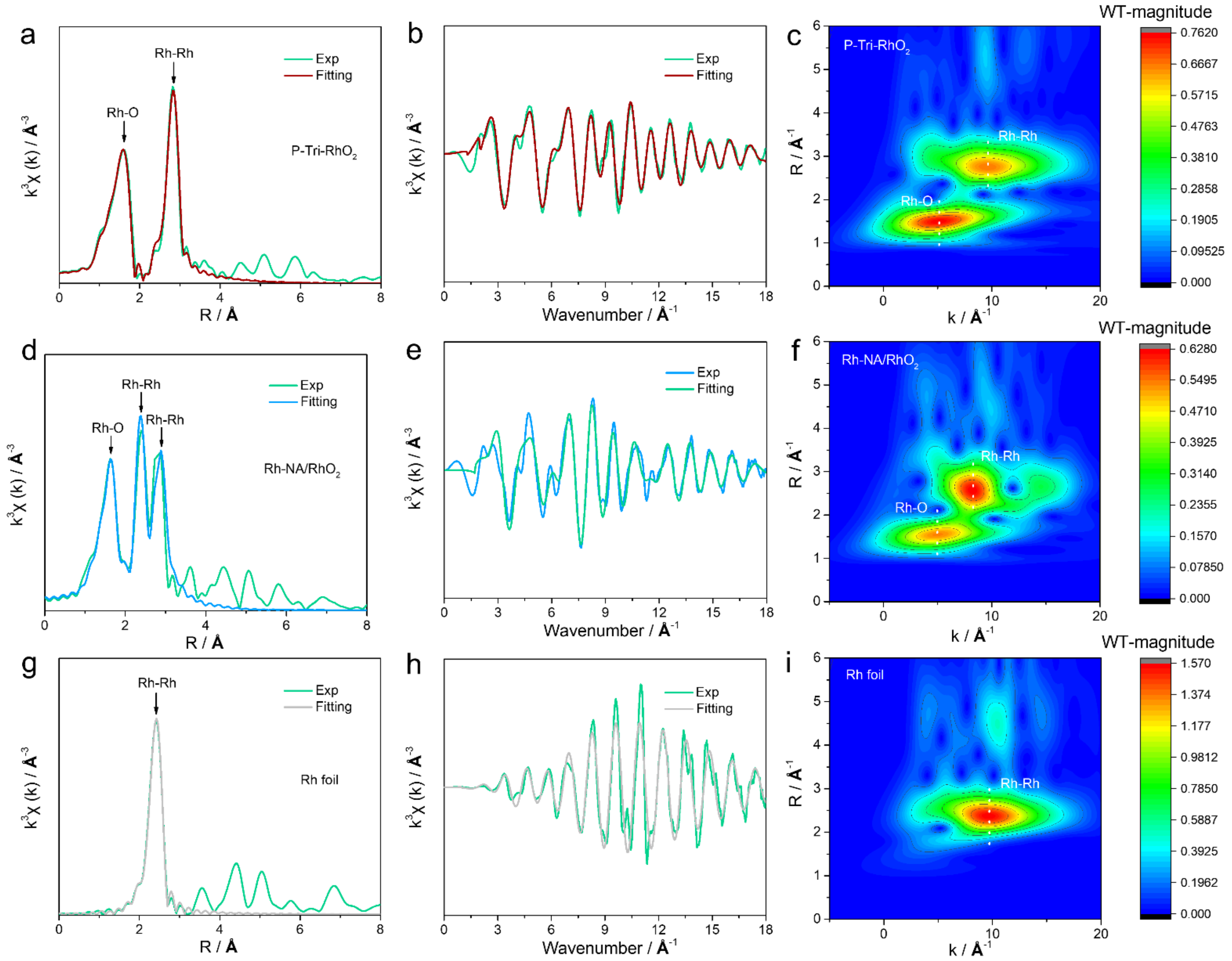

Supplementary Figure 8. **EXAFS fitting curves and wavelet transforms for P-Tri-RhO_2, Rh-NA/RhO_2 and Rh foil.** (**a**), (**d**), (**g**) EXAFS fitting curves of P-Tri-RhO_2, Rh-NA/RhO_2 and Rh foil at *R* space. (**b**), (**e**), (**h**) EXAFS fitting curves of P-Tri-RhO_2, Rh-NA/RhO_2 and Rh foil at *q* space. (**c**), (**f**), (**i**) Wavelet transforms of Rh K-edge EXAFS for P-Tri-RhO_2, Rh-NA/RhO_2 and Rh foil.

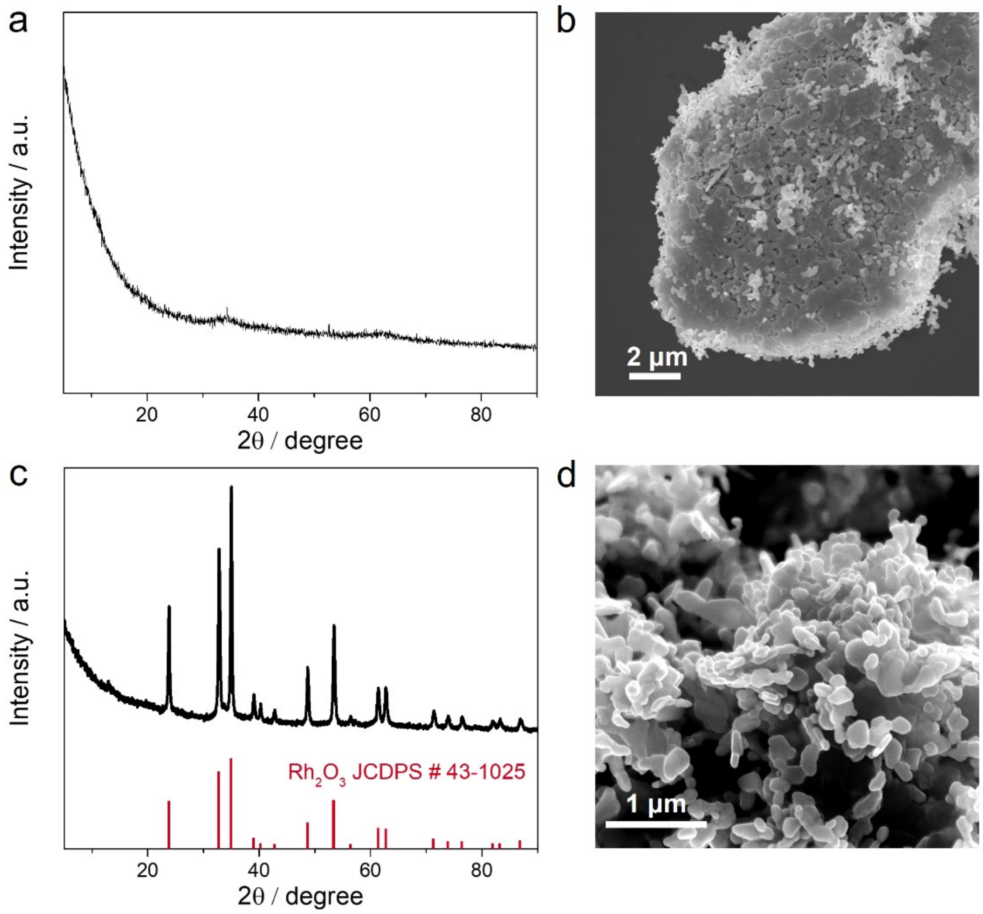

Supplementary Figure 9. **XRD patterns and SEM images obtained by changing the synthetic parameters.** (**a**) XRD pattern and (**b**) SEM image of the product obtained by directly mixing $RhCl_3$ and KOH. (**c**) XRD pattern and (**d**) SEM image of the product obtained by directly radio-frequency heating $RhCl_3$ without adding KOH.

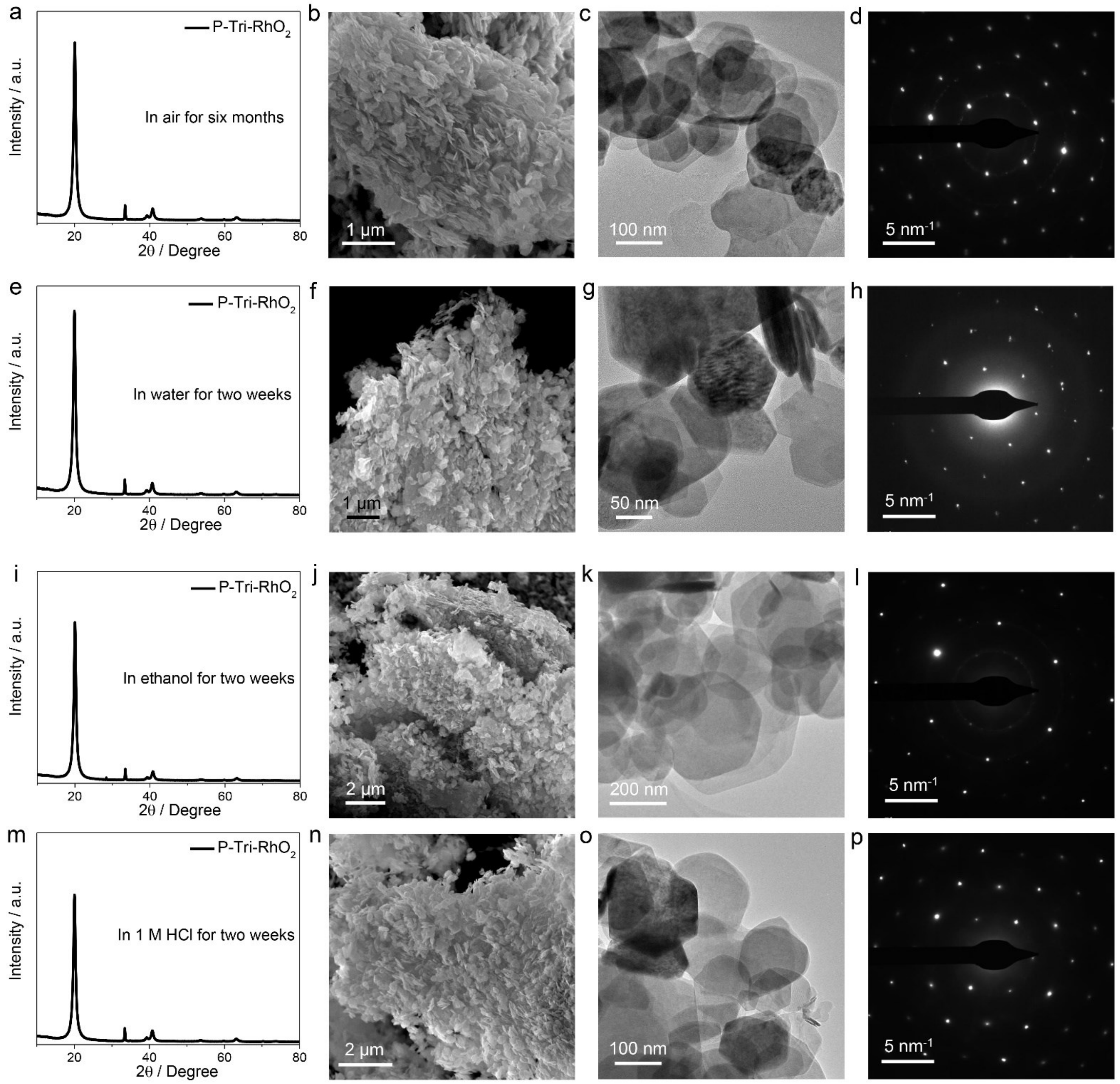

Supplementary Figure 10. The chemical stability for P-Tri-RhO$_2$. (**a-d**) The XRD pattern, SEM, TEM images and SAED pattern of P-Tri-RhO$_2$ after placing it in air for six months. (**e-h**) The XRD pattern, SEM, TEM images and SAED pattern of P-Tri-RhO$_2$ after soaking it in water for two weeks. (**i-l**) The XRD pattern, SEM, TEM images and SAED pattern of P-Tri-RhO$_2$ after soaking it in ethanol for two weeks. (**m-p**) The XRD pattern, SEM, TEM images and SAED pattern of P-Tri-RhO$_2$ after soaking it in 1 M hydrochloric acid solution for two weeks.

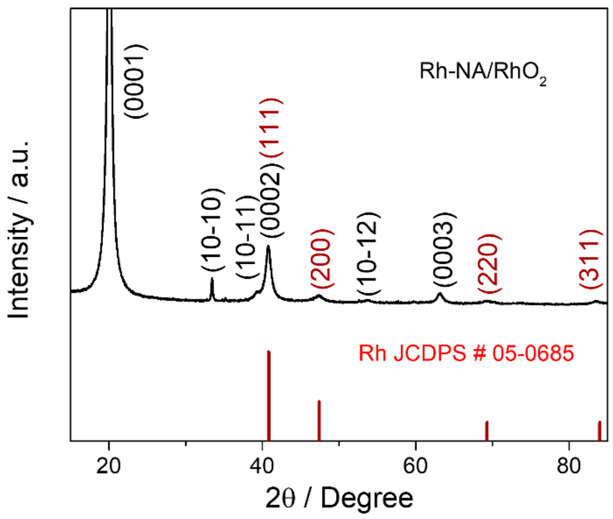

Supplementary Figure 11. XRD pattern of Rh-NA/RhO$_2$. The peaks of (111), (200), (220) and (311) planes of face-centered cubic Rh was detected and marked in red.

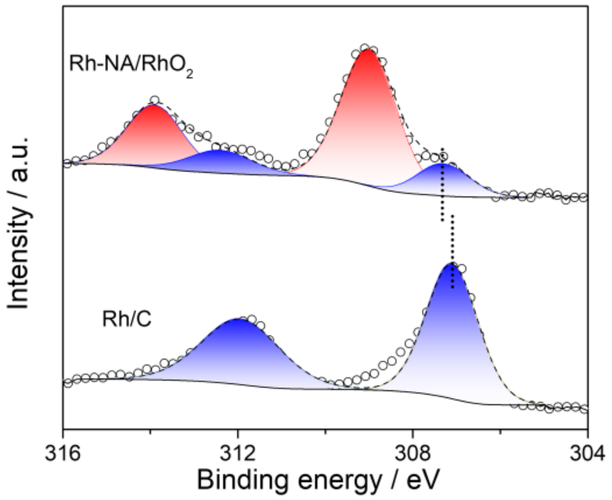

Supplementary Figure 12. XPS measurements for Rh-NA/RhO_2 and Rh/C. XPS spectra of Rh 3*d* peaks for Rh-NA/RhO_2 and Rh/C.

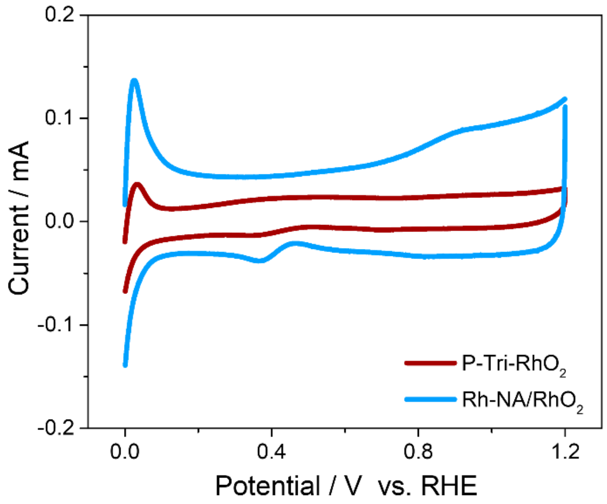

Supplementary Figure 13. Cyclic voltammetry (CV) tests for P-Tri-RhO$_2$ and Rh-NA/RhO$_2$. CV curves of P-Tri-RhO$_2$ and Rh-NA/RhO$_2$ in H_2-saturated 0.5 M H_2SO_4.

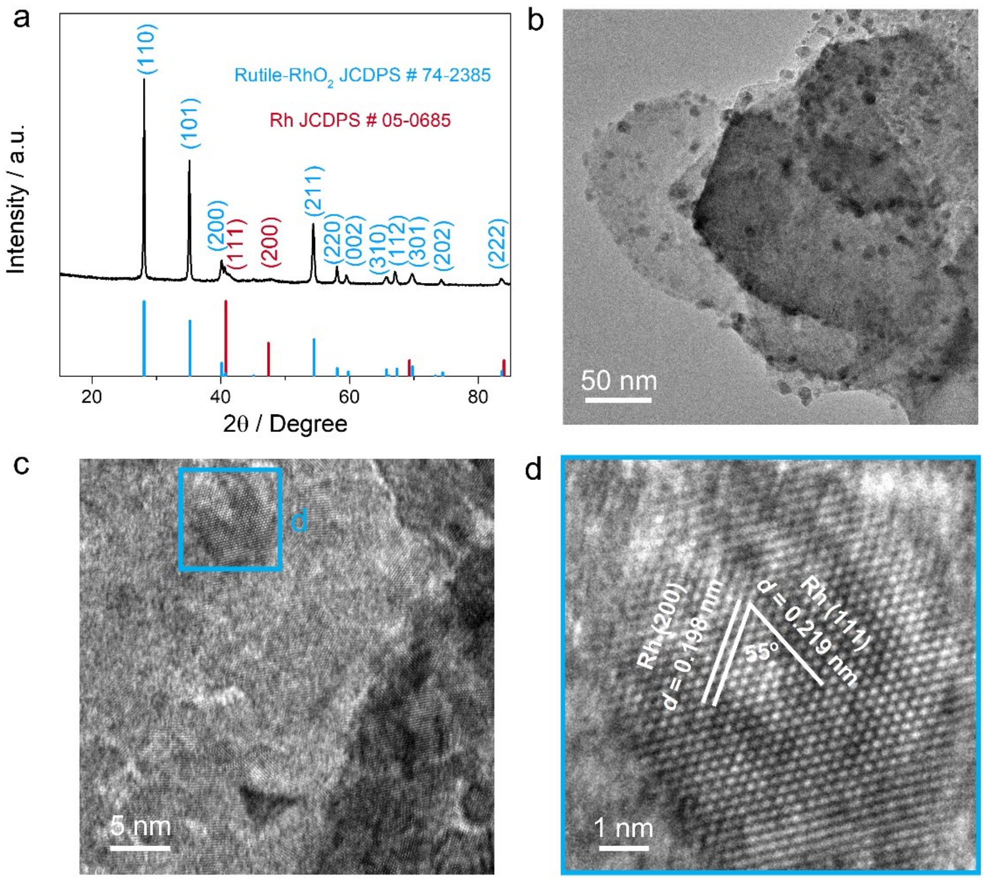

Supplementary Figure 14. **XRD pattern, TEM and HRTEM images of the product obtained by *in-situ* electroreduction of Rutile-RhO_2.** (**a**) XRD pattern of the product obtained by *in-situ* electroreduction of Rutile-RhO_2. (**b-d**) TEM and HRTEM images of the product obtained by *in-situ* electroreduction of Rutile-RhO_2.

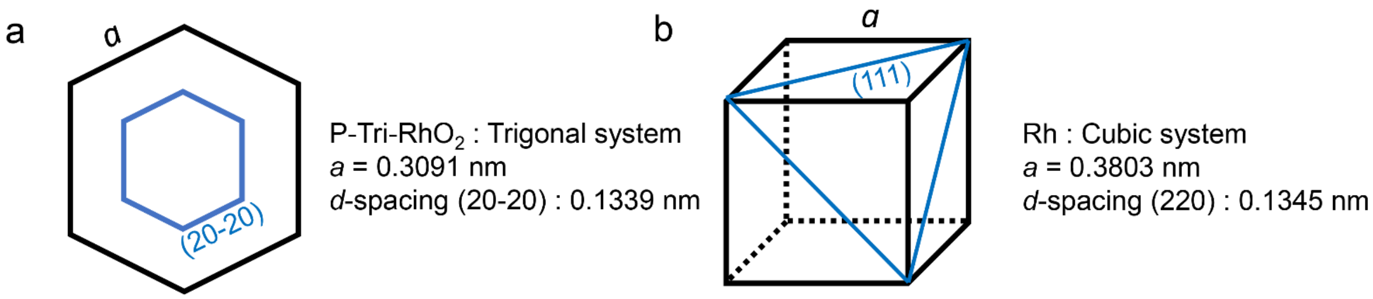

Supplementary Figure 15. Schematic views of crystal matching of Rh-NA/RhO_2 system. (**a**) The crystal structure information of P-Tri-RhO_2. (**b**) The crystal structure information of face-centered cubic Rh.

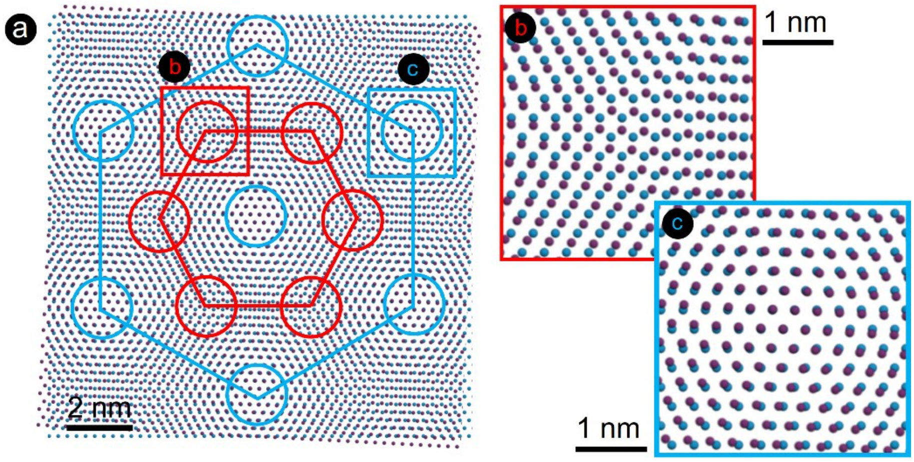

Supplementary Figure 16. Schematic views of Moire pattern obtained by twisting bilayer P-Tri-RhO_2 region with the rotation angle of 3º. (**a**) Simulated Moire pattern was obtained by twisting bilayer P-Tri-RhO_2 region with the rotation angle of 3º. The Rh atoms on different layers are marked with red and blue colors, respectively. (**b, c**) The enlarged areas from (**a**).

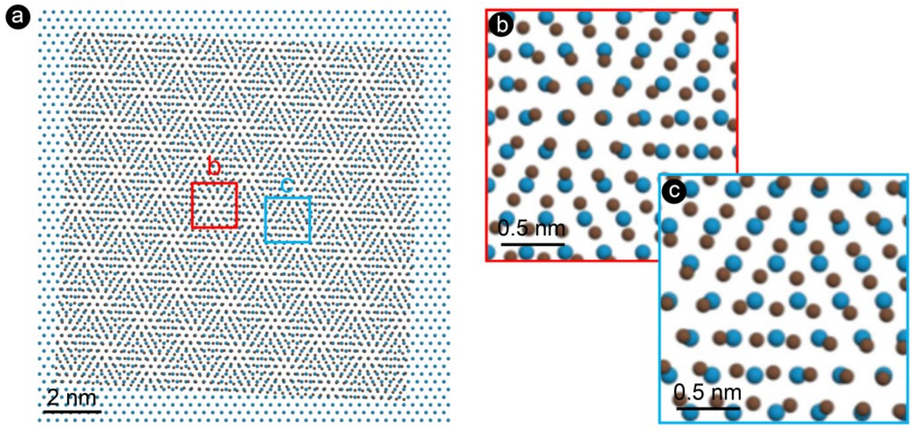

Supplementary Figure 17. Schematic views of Moire pattern obtained by twisting one single-layer P-Tri-RhO_2 and one single-layer metallic Rh with the rotation angle of 3º. Simulated Moire pattern was obtained by twisting one single-layer P-Tri-RhO_2 and one single-layer metallic Rh with the rotation angle of 3º. (**b, c**) The enlarged areas from (**a**).

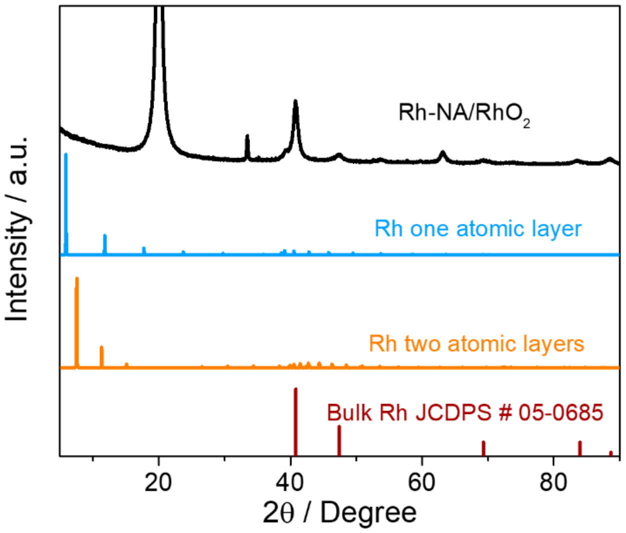

Supplementary Figure 18. Comparison of XRD patterns for Rh-NA/RhO_2 and different layers Rh. The XRD pattern of Rh-NA/RhO_2 and the simulated XRD patterns of different layers Rh.

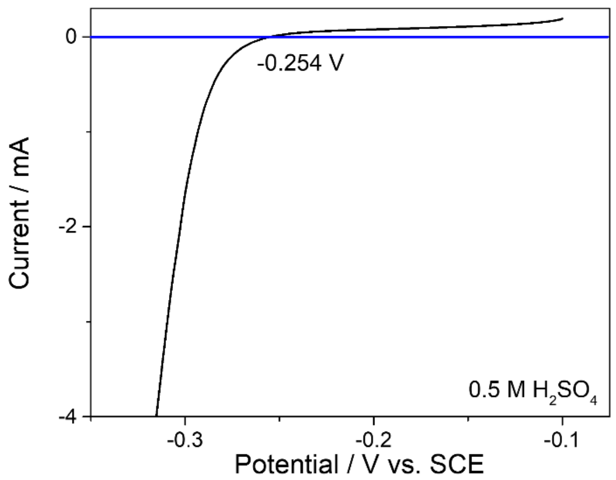

Supplementary Figure 19. Calibration of the saturated calomel electrode (SCE). Calibration of the SCE electrode with respect to reversible hydrogen electrode (RHE) in 0.5 M H_2SO_4 electrolyte bubbled with pure hydrogen gas at room temperature. Scan rate: 5 mV s^{-1}. Two Pt electrodes were used as the working and counter electrodes to calibrate the SCE.

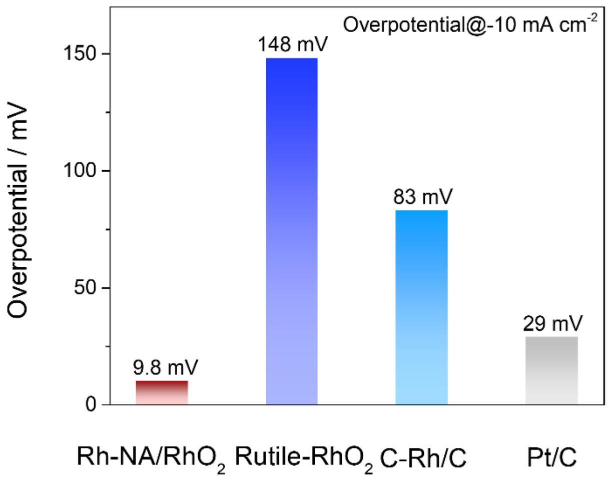

Supplementary Figure 20. Comparison of overpotentials for different catalysts. The overpotentials at the current density of -10 mA cm^{-2} for Rh-NA/RhO_2, Rutile-RhO_2, C-Rh/C and Pt/C.

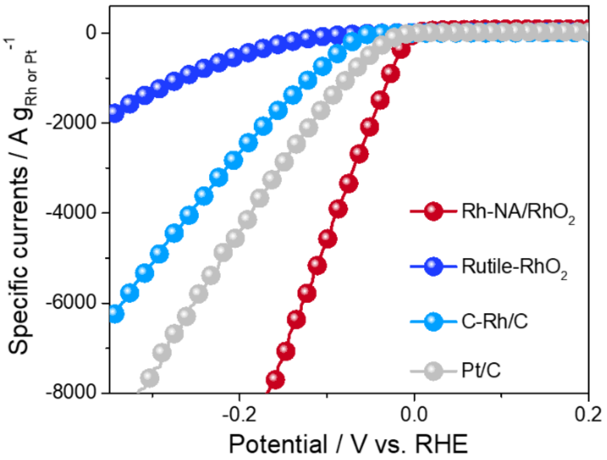

Supplementary Figure 21. The comparison of specific currents for different catalysts. The specific currents of Rh-NA/RhO_2, Rutile-RhO_2, C-Rh/C and Pt/C in 0.5 M H_2SO_4.

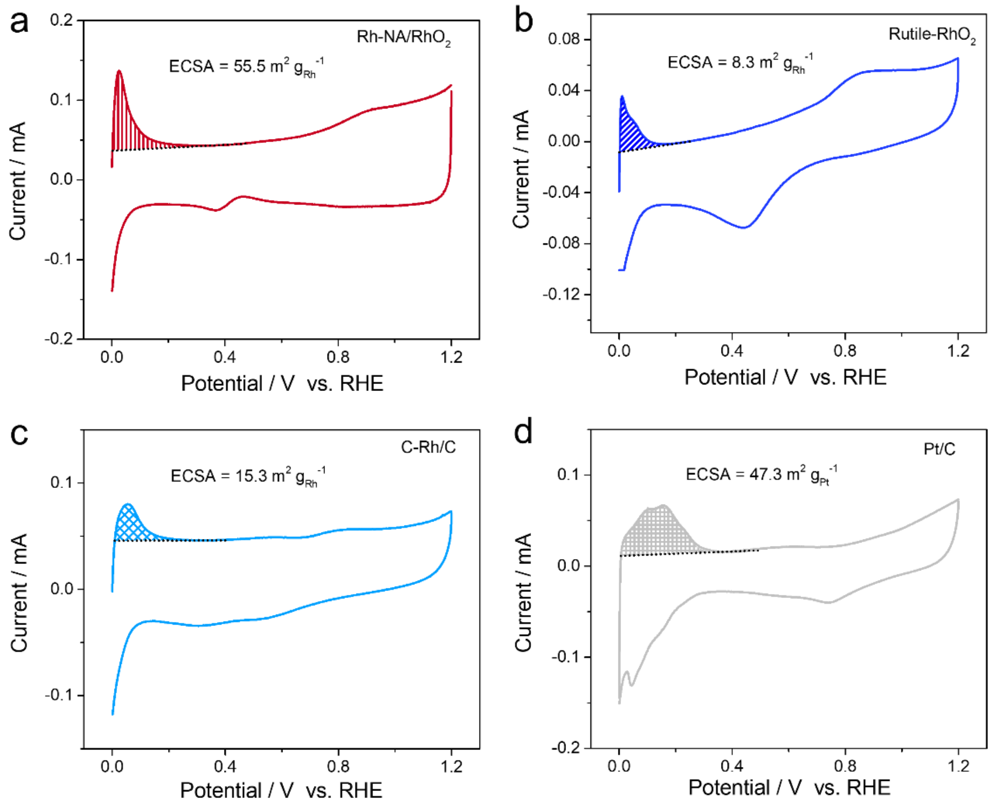

Supplementary Figure 22. The calculations of ECSA for different catalysts. CV curves of (**a**) Rh-NA/RhO_2, (**b**) Rutile-RhO_2, (**c**) C-Rh/C and (**d**) Pt/C in H_2-saturated 0.5 M H_2SO_4 with a scan rate of 50 mV s^{-1}.

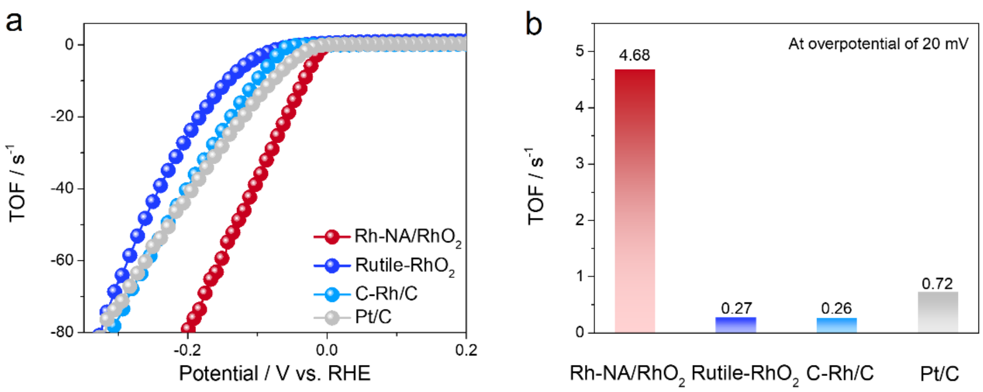

Supplementary Figure 23. The calculations of TOFs for different catalysts. (**a**) The comparison of TOFs for Rh-NA/RhO_2, Rutile-RhO_2, C-Rh/C and Pt/C at different potentials (vs. RHE). (**b**) The TOFs of Rh-NA/RhO_2, Rutile-RhO_2, C-Rh/C and Pt/C at the overpotential of 0.1 V.

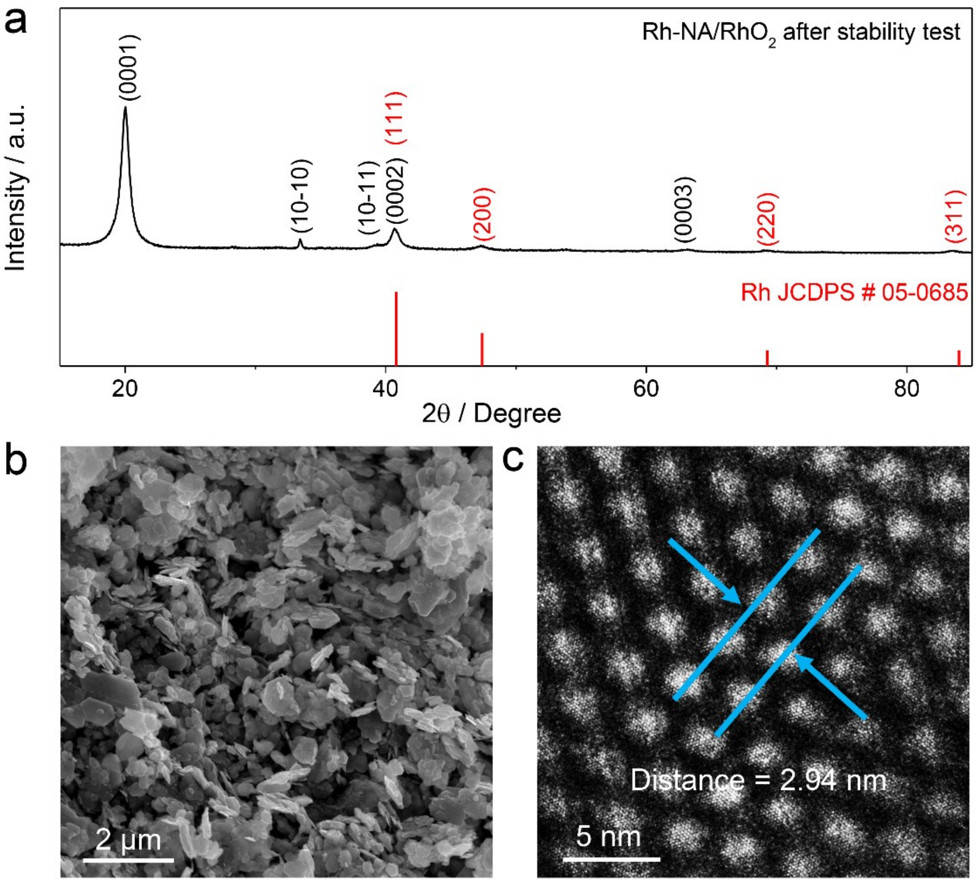

Supplementary Figure 24. **XRD pattern, SEM and STEM images of Rh-NA/RhO_2 after stability test.** (**a**) The XRD pattern of Rh-NA/RhO_2 after long-term HER stability test, where the (111), (200), (220) and (311) planes of face-centered cubic Rh was detected and marked in red. (**b, c**) SEM and STEM images of Rh-NA/RhO_2 after long-term HER stability test.

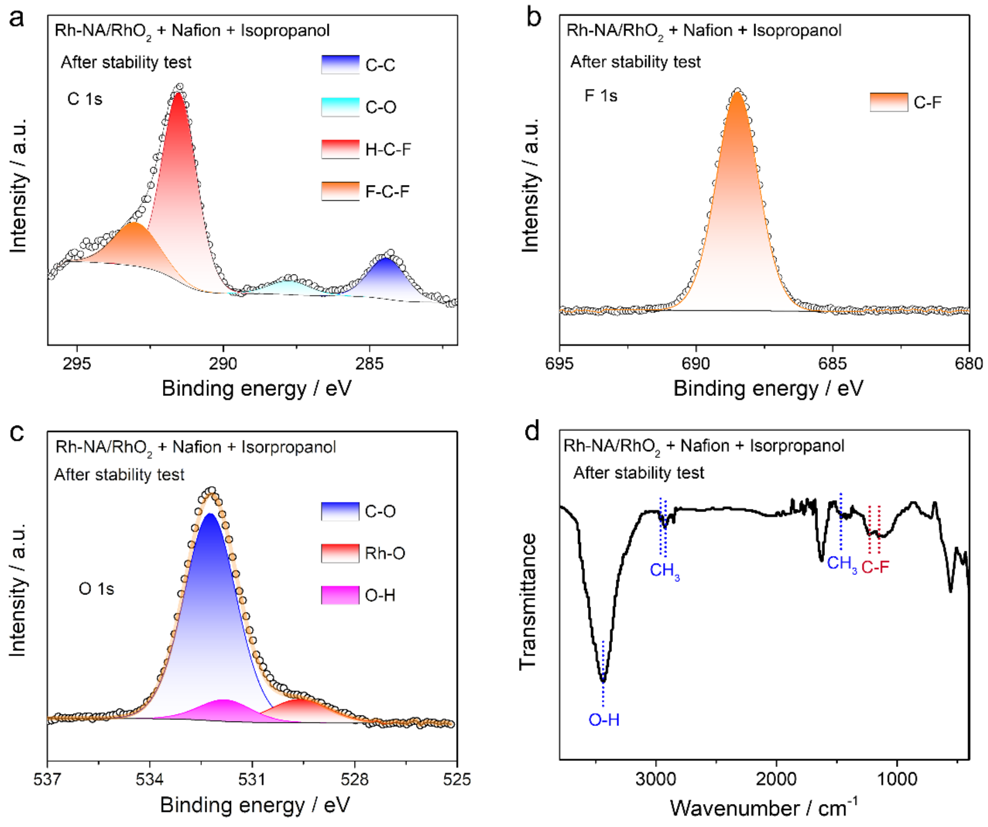

Supplementary Figure 25. XPS and FTIR measurements of Rh-NA/RhO_2 after long-term HER stability test. XPS spectra of (**a**) C 1s, (**b**) F 1s and (**c**) O 1s for Rh-NA/RhO_2 after long-term HER stability test. (**d**) FTIR spectrum of Rh-NA/RhO_2 after long-term HER stability test.

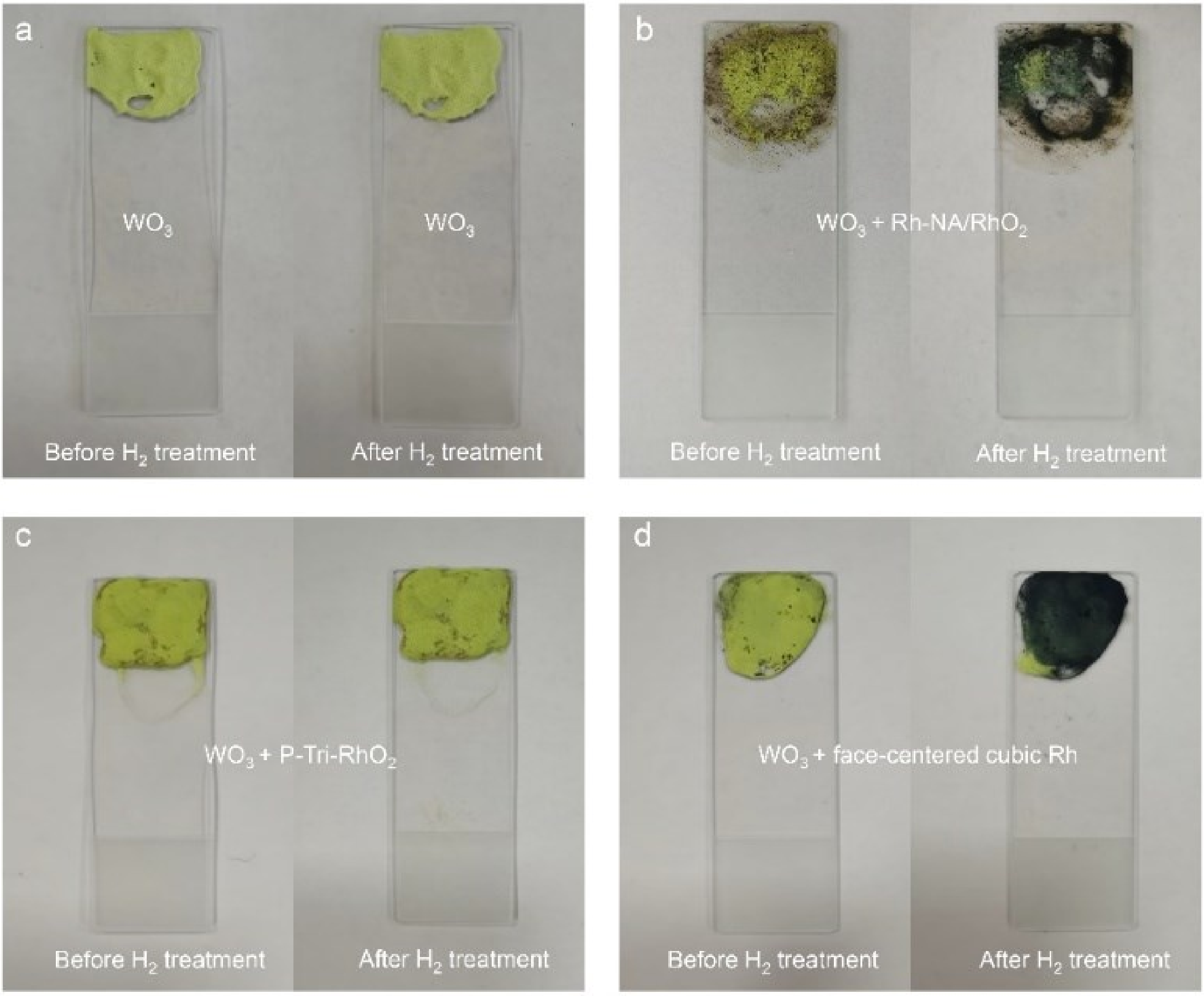

Supplementary Figure 26. The measurements of hydrogen spillover for Rh-NA/RhO_2. (**a**) The photographic image of WO_3 before and after H_2 treatment at room temperature. clearly showing no color change after H_2 treatment. (**b**) The photographic image of physical mixture for Rh-NA/RhO_2 and WO_3 before and after H_2 treatment at room temperature, clearly showing the color change of WO_3 from yellow to dark blue after H_2 treatment. (**c**) The photographic image of physical mixture P-Tri-RhO_2 and WO_3 before and after H_2 treatment at room temperature, clearly showing no color change after H_2 treatment. (**d**) The photographic image of physical mixture Rh and WO_3 before and after H_2 treatment at room temperature, clearly showing the color change of WO_3 from yellow to dark blue after H_2 treatment.

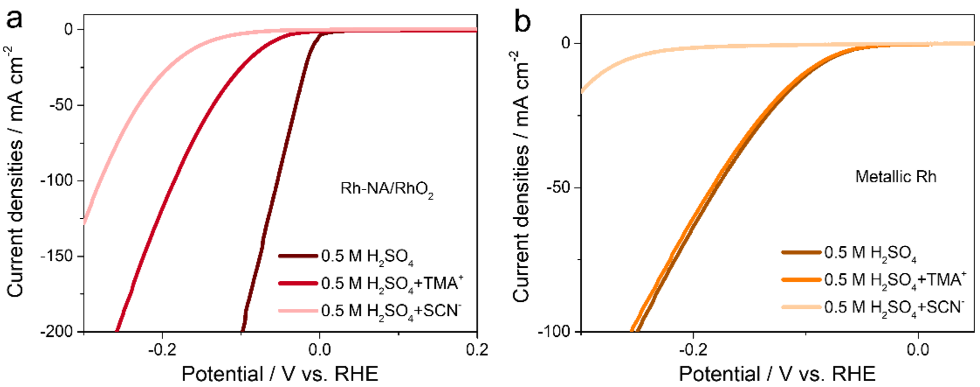

Supplementary Figure 27. Poison experiment for Rh-NA/RhO_2. Polarization curves for (**a**) Rh-NA/RhO_2 and (**b**) Rh before and after the addition of SCN^- or TMA^+ ions in 0.5 M H_2SO_4.

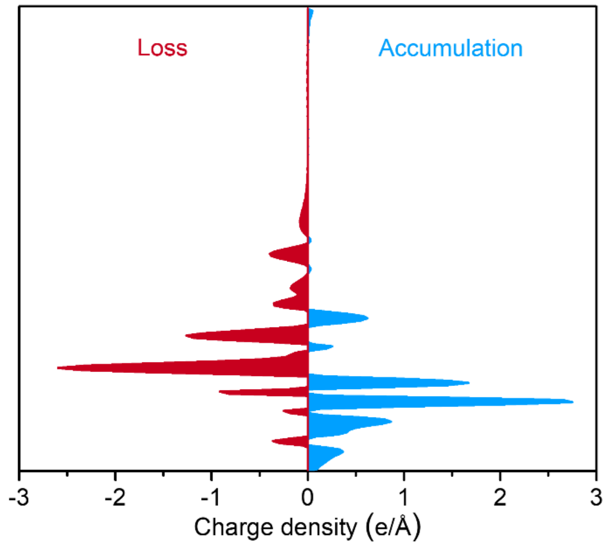

Supplementary Figure 28. Charge density difference of Rh-NA/RhO_2. Charge density difference of Rh-NA/RhO_2 along z direction.

Supplementary Tables

Supplementary Table 1. Crystallographic information for P-Tri-RhO_2 and Rutile-RhO_2.

**Material**	**Crystal system**	**Bravais lattice**	**Unit-cell dimensions**	**Space group**
**P-Tri-RhO_2**	**Trigonal**	**Primitive**	**$a = b = 3.091 \pm 0.004$ Å, $c = 4.407 \pm 0.004$ Å; $\alpha = \beta = 90°$, $\gamma = 120°$**	**P-3m1 (164)**
**Rutile-RhO_2**	Tetragonal	Primitive	$a = b = 4.489$ Å, $c = 3.090$ Å; $\alpha = \beta = \gamma = 90°$	$P4_2/mnm$ (136)

Supplementary Table 2. The hydrogen underpotential deposition-based area and BET values of P-Tri-RhO_2, Rh-NA/RhO_2, Rutile-RhO_2, C-Rh/C and Pt/C.

Catalyst	Hydrogen under potential deposition-based area / m^2 g^{-1}	BET area / m^2 g^{-1}
**P-Tri-RhO_2**	**8.8 ± 0.12**	**30.3 ± 0.82**
**Rh-NA/RhO_2**	**55.5 ± 1.13**	**/**
**Rutile-RhO_2**	8.3 ± 0.2	9.5± 0.25
**C-Rh/C**	15.3 ± 0.31	147.1 ± 3.01
**Pt/C**	47.3 ± 1.01	137.9 ± 2.82

Supplementary Table 3. The SDs of Rh atoms for different Moire patterns and Rh-NA/RhO_2.

Structure	The theoretical range of SD for different Moire patterns / nm^{-2}	The SDs for Rh-NA/RhO_2 / nm^{-2}
**Rh-NA/RhO_2**	/	**47.904 (theoretical) / 47.3±1.2 (actual)**
Moire pattern with two layer of P-Tri-RhO_2	12.086~24.172	/
Moire pattern with two layer of metallic Rh	15.969~31.938	/
Moire pattern with one layer of P-Tri-RhO_2 and one layer of metallic Rh	12.086~28.055	/

Supplementary Table 4. The HER activities of Rh-NA/RhO_2, Rutile-RhO_2, C-Rh/C and Pt/C in 0.5 M H_2SO_4*.

Catalyst	Overpotential @ 10 mA cm^{-2} / mV	Geometric activity / mA cm^{-2}	Mass activity / mA $mg_{Rh\ or\ Pt}^{-1}$	Tafel slope / mV dec^{-1}
**Rh-NA/RhO_2**	**9.8 ± 0.61**	**200 ± 4.0**	**4634 ± 30.0**	**24.0**
**Rutile-RhO_2**	148 ± 2.4	3 ± 0.1	67 ± 0.9	99.1
**C-Rh/C**	83 ± 1.6	17 ± 0.2	601 ± 5.2	43.7
**Pt/C**	29 ± 0.9	85 ± 1.8	1502 ± 12.8	30.0

*The geometric activity and mass activity were obtained at -0.1 V vs. RHE.

Supplementary Table 5. The exchange current density (j_0) of Rh-NA/RhO_2, Rutile-RhO_2, C-Rh/C and Pt/C in 0.5 M H_2SO_4.

Catalysts	Exchange current density / mA cm^{-2}
**Rh-NA/RhO_2**	**-3.356 ± 0.1258**
**Rutile-RhO_2**	-0.561 ± 0.0015
**C-Rh/C**	-0.142 ± 0.0003
**Pt/C**	-1.220 ± 0.0430

Supplementary Table 6. The comparison of HER activity performances of Rh-NA/RhO_2 with previous reported electrocatalysts.

Catalyst	Substrate	Electrolyte	Overpotential @ 10 mA cm^{-2} / mV	Tafel slope / mV dec^{-1}	Supplementary References
**Rh-NA/RhO_2**	**GCE**	**0.5 M H_2SO_4**	**9.8**	**24.0**	**This work**
**$PdCoO_2$**	GCE	1 M H_2SO_4	15	38	1
**Pt_1/OLC**	GCE	0.5 M H_2SO_4	38	36	2
**Pt–GT-1**	GCE	0.1 M $HClO_4$	18	24	3
**$Pd_3P_2S_8$**	GCE	0.5 M H_2SO_4	91	29	4
**Ru@C_2N**	GCE	0.5 M H_2SO_4	13.5	30	5
**Pt-SAs/TMDs**	GCE	0.5 M H_2SO_4	32	28	6
**RuIr-NC**	RDE	0.5 M H_2SO_4	46	32	7
**1%Pd–MoS_2/CP**	GCE	0.5 M H_2SO_4	78	32	8
**Rh_2P**	GCE	0.5 M H_2SO_4	14	31.7	9
**Rh NS/C**	GCE	0.1 M $HClO_4$	15.8	61.5	10
**Au–Rh**	RDE	0.5 M H_2SO_4	20	29	11
**Rh-MoS_2**	RDE	0.5 M H_2SO_4	67	54	12
**Rh_2S_3–ThickHNP/C**	GCE	0.5 M H_2SO_4	88	44	13
**a-$RuTe_2$ PNRs**	GCE	0.5 M H_2SO_4	33	35	14
**RuCu NPs**	GCE	0.5 M H_2SO_4	19	26	15
**Pt-PVP/TNR@GC**	RDE	0.5 M H_2SO_4	21	27	16
**PdCu/Ir**	GCE	0.1 M $HClO_4$	20	59.6	17

**$RuIrO_x$**	RDE	0.5 M H_2SO_4	12	23	18
**Pt_5/HMCS**	RDE	0.5 M H_2SO_4	20.7	28.3	19
**AL-Pt/Pd_3Pb**	GCE	0.5 M H_2SO_4	13.8	18	20
**Pt-AC/DG**	GCE	0.1 M $HClO_4$	21	27.5	21

Supplementary Table 7. The comparison of minimum cluster size and minimum interparticle distance with previous reported materials.

Materials	Minimum cluster size	Minimum inter-particle distance	Minimum inter-surface distance	Preparation method	Supplementary References
**Rh-NA/RhO_2**	**3.2 nm**	**3.709 nm**	**0.5 nm**	**In situ formed Rh nanoarrays on metastable trigonal phase RhO_2 nanosheets**	**This work**
**As_2Se_3-PVDF core-shell**	~200 nm	~800 nm	~1 μm	Melting commercially bought glasses	22
**Fe_3O_4 NCA**	~8 nm	~10.7 nm	~3 nm	Controlled thermal decomposition	23
**SHNO arrays**	50 nm	100 nm	~115 nm	Ultra-high vacuum magnetron sputtering machine	24
**3D gold nanostructures**	5.8 nm	7.1 nm	~3 nm	Pressure-directed assembly (PDA)	25
**Platinum nanoparticle array**	40 nm	150 nm	~100 nm	Electron beam lithography (EBL)	26
**Au@Ag core-shell**	17 nm	45 nm	~30 nm	Block copolymer (BCP) lithography	27
**Nanopattern apertures**	12 nm	100 nm	33 nm	Focused-ion-beam-guided prepatterning technique	28
**AgNC array**	100 nm	2000 nm	1.8 μm	Directed assembly of silver nanocubes (AgNCs) onto TiO_2 coated Si substrates.	29
**Ag nanoparticle arrays**	200 nm	540 nm	~296 nm	Thermal evaporation and focused-ion beam	30
**Pt nanoparticle (NP) arrays**	/	57 nm	~25 nm	Electrochemical anodization process	31
**$Au_{0.5}Pt_{0.5}$ alloy**	6 nm	20 nm	~26 nm	Block copolymer micellar nanoLithography (BCML)	32
**Si_3N_4**	15 nm	100 nm	~73.5 nm	EUV interference lithography	33
**Platinum**	28 nm	100 nm	~100 nm	Electron beam	34

**nanoparticle array**				lithography (EBL)	
**Cu NPs arrays**	110 nm	350 nm	~206 nm	Interfacial self-assembly	35
**Fe_2O_3 nanoparticle array**	9.01 nm	19.23 nm	~16.3 nm	Block copolymer lithography	36
**Au nanoparticle arrays**	2.4 nm	26.8 nm	~24.4 nm	Ar plasma treatment	37
**Pt nanoparticle arrays**	2.2 nm	10.8 nm	~23.4 nm	PS-b-P2VP diblock-copolymers	38
**$Si_{0.5}CeO_{0.5}$**	5 nm	10 nm	~7 nm	Co-self-assembling route	39
**Au nanoparticles**	5.8 nm	10.5 nm	~2.1 nm	Asymmetric heating of a novel precursor	40
**Pt Nanoparticle Arrays**	20 nm	150 nm	~135 nm	Electron beam lithography (EBL)	41
**Pt nanoparticle arrays**	28 nm	100 nm	~162 nm	Electron beam lithography (EBL)	42
**Platinum cluster arrays**	50 nm	200 nm	~73.7 nm	Electron beam lithography (EBL)	43

Supplementary Notes

Supplementary Note 1. The calculation of surface densities (SDs) of Rh atoms for different Moire patterns and Rh-NA/RhO_2.

1. For Moire patterns consisting of two single-layer P-Tri-RhO_2

For a single-layer P-Tri-RhO2, the surface density of Rh atom is:

$$SD = 1 \div \left(\frac{\sqrt{3}}{2} \times 0.3091^2\ nm^2\right) = 12.086\ nm^{-2}$$

If Moire patterns were obtained by twisting two single-layer P-Tri-RhO_2 with different rotation angles, the maximum surface density was determined to be $\leq 2 \times 12.086 = 24.172\ nm^{-2}$. So the range of surface density for Moire patterns obtained by twisting bilayer P-Tri-RhO_2 was determined to be $12.086\ nm^{-2} \leq SD \leq 24.172\ nm^{-2}$.

2. For Moire patterns consisting of two single-layer metallic Rh

For a single-layer metallic Rh, the surface density of Rh atom is:

$$SD = 1 \div \left(\frac{\sqrt{3}}{2} \times 0.2689^2\ nm^2\right) = 15.969\ nm^{-2}$$

If Moire patterns were obtained by twisting two single-layer metallic Rh with different rotation angles, the maximum surface density was determined to be $2 \times 15.969 = 31.938\ nm^{-2}$. So the range of surface density for Moire patterns obtained by twisting bilayer face-centered cubic Rh was determined to be $15.969\ nm^{-2} \leq SD \leq 31.938\ nm^{-2}$.

3. For Moire patterns consisting of one single-layer P-Tri-RhO_2 and one single-layer metallic Rh

If Moire patterns were obtained by twisting one single layer P-Tri-RhO_2 and one single layer metallic Rh with different rotation angles, the maximum surface density was determined to be $15.969 + 12.086 = 28.055\ nm^{-2}$. So the range of surface density for Moire patterns obtained by twisting P-Tri-RhO_2 and metallic Rh was determined to be $12.086\ nm^{-2} \leq SD \leq 28.055\ nm^{-2}$.

4. For face-centered cubic Rh nanocrystal arrays

The theoretical surface density of rhodium atoms for face-centered cubic Rh along the [111] growth direction.

$$SD = 3 \div \left(\frac{\sqrt{3}}{2} \times 0.2689^2\ nm^2\right) = 47.904\ nm^{-2}$$

5. The actual SD of Rh atoms in Rh-NA/RhO_2 counted from the (scanning transmission electron microscopy) STEM image (Fig. 2f)

As shown in **Fig. 2f**, the actual SD of Rh atoms in Rh-NA/RhO_2 is counted to be $47.3\pm1.2\ nm^{-2}$.

Supplementary Note 2. Proposed H spillover assisted HER mechanism for Rh-NA/RhO_2.

Based on the simulation, a possible path is proposed to explain the electrocatalysis of Rh-NA/RhO_2. HER may take place on any Rh nanoparticles by themselves and with the help of H spillover from Rh nanoparticle to contact interface between Rh nanoparticle and P-Tri-RhO_2 substrate.

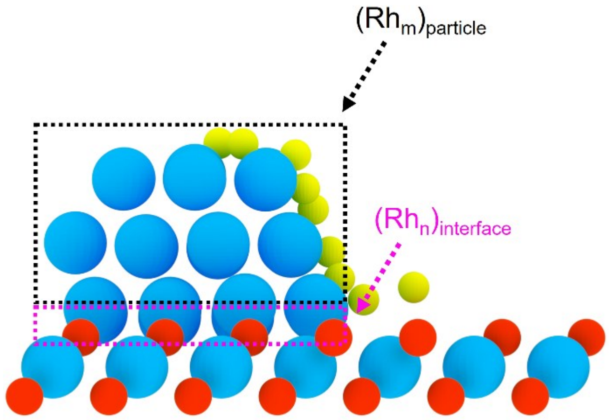

Assuming that H spillover is from Rh nanoparticle $(Rh_m)_{particle}$ to contact interface $(Rh_n)_{interface}$, the first step is the adsorption of H atom on the surface of Rh nanoparticle $(Rh_m)_{particle}$,

$$(Rh_m)_{particle} - (Rh_n)_{interface} + H^+ + e^- \Leftrightarrow H - (Rh_m)_{particle} - (Rh_n)_{interface} \quad \text{(S1)}$$

Secondly, the spillover of H atom from $(Rh_m)_{particle}$ to $(Rh_n)_{interface}$,

$$H - (Rh_m)_{particle} - (Rh_n)_{interface} \Leftrightarrow (Rh_m)_{particle} - (Rh_n)_{interface} - H \quad \text{(S2)}$$

Thirdly, the Heyrovsky process on the interface of Rh nanoparticle and P-Tri-RhO_2 $(Rh_n)_{interface}$,

$$(Rh_m)_{particle} - (Rh_n)_{interface} - H + H^+ + e^- \rightarrow H_2 + (Rh_m)_{particle} - (Rh_n)_{interface} \quad \text{(S3)}$$

The kinetics may be complicated. The reaction velocity of hydrogen evolution may be written as $r = k_3\theta_{(Rh_n)_{interface}-H}C_{H^+}$, where r is the reaction rate; k the rate constant; θ the fractional occupancy of the H-adsorption sites; and C_{H^+} the concentration of hydrogen ion.

In the steady state, for $\theta_{(Rh_n)_{interface}-H}$,

$$\frac{d\theta_{(Rh_n)_{interface}-H}}{dt} =$$

$$k_2\theta_{(Rh_m)_{particle}-H}(1 - \theta_{(Rh_n)_{interface}-H}) - k_{-2}\theta_{(Rh_n)_{interface}-H}(1 - \theta_{(Rh_m)_{particle}-H}) -$$

$$k_3\theta_{(Rh_n)_{interface}-H}C_{H^+};$$

and for $\theta_{(Rh_m)_{particle}-H}$

$$\frac{d\theta_{(Rh_m)_{particle}-H}}{dt} = k_1(1-\theta_{(Rh_m)_{particle}-H})C_{H^+} - k_{-1}\theta_{(Rh_m)_{particle}-H} - k_2\theta_{(Rh_m)_{particle}-H}(1 - \theta_{(Rh_n)_{interface}-H}) + k_{-2}\theta_{(Rh_n)_{interface}-H}(1-\theta_{(Rh_m)_{particle}-H}).$$

At low overpotential,

$$\theta_{(Rh_n)_{interface}-H} \approx \frac{k_2\theta_{(Rh_m)_{particle}-H}}{k_2\theta_{(Rh_m)_{particle}-H} + k_{-2} - k_{-2}\theta_{(Rh_m)_{particle}-H} + k_3C_{H^+}} \approx \frac{k_{20}}{k_{-20}}\theta_{(Rh_m)_{particle}-H}e^{-\frac{F\Delta\phi}{RT}}$$

$$\theta_{(Rh_m)_{particle}-H} \approx \frac{k_1C_{H^+} + k_{-2}\theta_{(Rh_n)_{interface}-H}}{k_1C_{H^+} + k_{-1} + k_2 + k_{-2}\theta_{(Rh_n)_{interface}-H} - k_2\theta_{(Rh_n)_{interface}-H}} \approx \frac{k_{10}}{k_{-10}}C_{H^+}e^{-\frac{F\Delta\phi}{RT}}$$

Then, $r = k_3\theta_{(Rh_n)_{interface}-H}C_{H^+} = \frac{k_{10}k_{20}k_{30}}{k_{-10}k_{-20}}C_{H^+}^2e^{-\frac{(2+\alpha)F\Delta\phi}{RT}}$ (S4)

And $-j = Fr = \frac{k_{10}k_{20}k_{30}}{k_{-10}k_{-20}}FC_{H^+}^2e^{-\frac{(2+\alpha)F\Delta\phi}{RT}}$

$lg(-j) = Const + 2\,lg\,C_{H^+} - \frac{(2+\alpha)F}{2.303RT}\Delta\phi$ (S5)

Therefore, the Tafel slope is: $\frac{2.303RT}{(2+\alpha)F}$ = 0.024 V dec^{-1} (assuming $\alpha = 0.5$).

Supplementary References

1. Podjaski, F. et al. Rational strain engineering in delafossite oxides for highly efficient hydrogen evolution catalysis in acidic media. *Nat. Catal*. **3**, 55-63 (2020).
2. Li, D. B. et al. Atomically dispersed platinum supported on curved carbon supports for efficient electrocatalytic hydrogen evolution. *Nat. Energy* **4**, 512-518 (2019).
3. Tiwari, J. N. et al. Multicomponent electrocatalyst with ultralow Pt loading and high hydrogen evolution activity. *Nat. Energy* **3**, 773-782 (2018).
4. Zhang, X. et al. Lithiation-induced amorphization of $Pd_3P_2S_8$ for highly efficient hydrogen evolution. *Nat. Catal*. **1**, 460-468 (2018).
5. Mahmood, J. et al. An efficient and pH-universal ruthenium-based catalyst for the hydrogen evolution reaction. *Nat. Nanotechnol*. **12**, 441-446 (2017).
6. Shi, Y. et al. Electronic metal-support interaction modulates single-atom platinum catalysis for hydrogen evolution reaction. *Nat. Commun*. **12**, 3021 (2021).
7. Wu, D. S. et al. Efficient overall water splitting in acid with anisotropic metal nanosheets. *Nat. Commun*. **12**, 1145 (2021).
8. Luo, Z. Y. et al. Chemically activating MoS_2 via spontaneous atomic palladium interfacial doping towards efficient hydrogen evolution. *Nat. Commun*. **9**, 2120 (2018).
9. Yang, F. L. et al. A monodisperse Rh_2P-based electrocatalyst for highly efficient and pH-universal hydrogen evolution reaction. *Adv. Energy Mater*. **8**, 1703489 (2018).
10. Wang, K. et al. Wrinkled Rh_2P nanosheets as superior pH-Universal electrocatalysts for hydrogen evolution catalysis. *Adv. Energy Mater*. **8**, 1801891 (2018).
11. Du, R. et al. Engineering multimetallic aerogels for pH-universal HER and ORR electrocatalysis. *Adv. Energy Mater*. **10**, 1903857 (2020).

12. Meng, X. Y. et al. Distance synergy of MoS_2-confined rhodium atoms for highly efficient hydrogen evolution. *Angew. Chem. Int. Ed.* **59**, 10502-10507 (2020).

13. Yoon, D. et al. Facet-controlled hollow Rh_2S_3 hexagonal nanoprisms as highly active and structurally robust catalysts toward hydrogen evolution reaction. *Energy Environ. Sci.* **9**, 850-856 (2016).

14. Wang, J. et al. Amorphization activated ruthenium-tellurium nanorods for efficient water splitting. *Nat. Commun.* **10**, 5692 (2019).

15. Yao, Q. et al. Channel-rich RuCu nanosheets for pH-universal overall water splitting electrocatalysis. *Angew. Chem. Int. Ed.* **58**, 13983-13988 (2019).

16. Li, C. et al. Polyvinylpyrrolidone-coordinated single-site platinum catalyst exhibits high activity for hydrogen evolution reaction. *Angew. Chem. Int. Ed.* **59**, 15902-15907 (2020).

17. Li, M. G. et al. Exclusive strain effect boosts overall water splitting in PdCu/Ir core/shell nanocrystals. *Angew. Chem. Int. Ed.* **60**, 8243-8250 (2021).

18. Zhuang, Z. W. et al. Three-dimensional open nano-netcage electrocatalysts for efficient pH-universal overall water splitting. *Nat. Commun.* **10**, 4875 (2019).

19. Wan, X. K., Wu, H. B., Guan, B. Y., Luan, D. Y. & Lou, X. W. Confining sub-nanometer Pt clusters in hollow mesoporous carbon spheres for boosting hydrogen evolution activity. *Adv. Mater.* **32**, 1901349 (2019).

20. Yao, Y. C. et al. Engineering the electronic structure of submonolayer Pt on intermetallic Pd_3Pb via charge transfer boosts the hydrogen evolution reaction. *J. Am. Chem. Soc.* **141**, 19964-19968 (2019).

21. Chen, Q. Q. et al. Carbon-defect-driven electroless deposition of Pt atomic clusters for highly efficient hydrogen evolution. *J. Am. Chem. Soc.* **142**, 5594-5601 (2020).

22. Yaman, M. et al. Arrays of indefinitely long uniform nanowires and nanotubes. *Nat. Mater.* **10**, 494-501 (2011).

23. Ong, W. L., Rupich. S. M. Talapin, D. V. McGaughey, A. J. H. & Malen, J. A. Surface chemistry mediates thermal transport in three-dimensional nanocrystal arrays. *Nat. Mater.* **12**, 410-415 (2013).

24. Zahedinejad, M. et al. Two-dimensional mutually synchronized spin Hall nano-oscillator arrays for neuromorphic computing. *Nat. Nanotechnol*. **15**, 47-52 (2019).

25. Wu, H. et al. Nanostructured gold architectures formed through high pressure-driven sintering of spherical nanoparticle arrays. *J. Am. Chem. Soc.* **132**, 12826-12828 (2010).

26. Baldelli, S., Eppler, A. S., Anderson, E., Shen, Y. R. & Somorjai, G. A. Surface enhanced sum frequency generation of carbon monoxide adsorbed on platinum nanoparticle arrays. *J. Chem. Phys.* **113**, 5432-5438 (2000).

27. Cha, S. K. et al. Au-Ag core-shell nanoparticle array by block copolymer lithography for synergistic broadband plasmonic properties. *ACS Nano* **9**, 5536-5543 (2015).

28. Lin, M. N. et al. Fabrication of an ordered nanoparticle array with a nanoaperture membrane used as a contact-mask. *Nanotechnology* **17**, 315-319 (2006).

29. Speckbacher, M. et al. Directed assembly of nanoparticle threshold-selector arrays. *Adv. Electron. Mater.* **5**, 1900098 (2019).

30. Huang, X. D., Lou, C. G. & Zhang, H. Experimentally demonstrating plasmonic lattice mode in periodic Ag nanoparticle arrays on quartz trapezoidal pillars. *J. Phys. D Appl. Phys.* **51**, 465101 (2018).

31. Owusu-Ansah, E., Horwood, C. A., El-Sayed, H. A., Birss, V. I. & Shi, Y. J. J. A method for the formation of Pt metal nanoparticle arrays using nanosecond pulsed laser dewetting. *Appl. Phys. Lett.* **106**, 203103 (2015).

32. Jahn, S., Lechner, S. J., Freichels, H., Moller, M. & Spatz, J. P. Precise Au_xPt_{1-x} alloy nanoparticle array of tunable composition for catalytic applications. *Sci. Rep.* **6**, 20536 (2016).

33. Karim, W. et al. High-resolution and large-area nanoparticle arrays using EUV interference lithography. *Nanoscale* **7**, 7386-7393 (2015).

34. Zhu, J. & Somorjai, G. A. Formation of platinum silicide on a platinum nanoparticle array model catalyst deposited on silica during chemical reaction. *Nano Lett.* **1**, 8-13 (2001).

35. Hang, L. F. et al. Copper nanoparticle@graphene composite arrays and their enhanced catalytic performance. *Acta Mater*. **105**, 59-67 (2016).

36. Shi, D. O. et al. Sub-nanometer level size tuning of a monodisperse nanoparticle array via block copolymer lithography. *Adv. Funct. Mater*. **21**, 250-254 (2011).

37. Kumar, S. & Zou, S. Z. Electrooxidation of CO on uniform arrays of Au nanoparticles: effects of particle size and interparticle spacing. *Langmuir* **25**, 574-581 (2009).

38. Yang, H. Z., Kumar, S. & Zou, S. Z. Electroreduction of O_2 on uniform arrays of Pt nanoparticles. *J. Electroanal. Chem*. **688**, 180-188 (2013).

39. Chane-Ching, J. Y. et al. Surface characterization and properties of ordered arrays of CeO_2 nanoparticles embedded in thin layers of SiO_2. *Langmuir* **21**, 1568-1574 (2005).

40. Egusa, S. Redmond, P. L. & Scherer, N. F. Thermally-driven nanoparticle array growth from atomic Au precursor solutions. *J. Phys. Chem. C* **111**, 17993-17996 (2007).

41. Eppler, A. S., Rupprechter, G., Anderson, E. A. & Somorjai, G. A. Thermal and chemical stability and adhesion strength of Pt nanoparticle arrays supported on silica studied by transmission electron microscopy and atomic force microscopy. *J. Phys. Chem. B* **104**, 7286-7292 (2000).

42. Eppler, A. S., Zhu, J., Anderson, E. A. & Somorjai, G. A. Model catalysts fabricated by electron beam lithography: AFM and TPD surface studies and hydrogenation/dehydrogenation of cyclohexene plus H_2 on a Pt nanoparticle array supported by silica. *Top. Catal*. **13**, 33-41 (2000).

43. Jacobs, P. W., Ribeiro, F. H., Somorjai, G. A. & Wind, S. J. New model catalysts: Uniform platinum

cluster arrays produced by electron beam lithography. *Catal. Lett*. **37**, 131-136 (1996).
