# Supplementary material for: Coupling of nanocrystal hexagonal array and two-dimensional metastable substrate boosts H2-production
Source: Nat Commun. 2022 Oct 3;13:5828. doi: 10.1038/s41467-022-33512-5 (PMC9530234; doi:10.1038/s41467-022-33512-5)
Supplement: Supplementary file 3 — Description of Additional Supplementary Files [file 41467_2022_33512_MOESM3_ESM.pdf]

Description of Additional Supplementary Files

File Name: Supplementary Movie 1
Description: The movie of the simulated Morie pattern by twisting bilayer P-Tri-RhO_2.
